# An Exploration of Motion‐Sampling Interactions in 3D MRI for Neuroimaging

**DOI:** 10.1002/mrm.70132

**Published:** 2025-11-07

**Authors:** Sophie Schauman, Adam van Niekerk, Ola Norbeck, Henric Rydén, Enrico Avventi, Stefan Skare

**Affiliations:** ^1^ Department of Clinical Neuroscience Karolinska Institutet Stockholm Sweden; ^2^ Department of Neuroradiology Karolinska University Hospital Stockholm Sweden

**Keywords:** 3D, artefacts, k‐space, motion, sampling, view order

## Abstract

**Purpose:**

To investigate how rigid head motion interacts with 3D MRI k‐space sampling strategies and to introduce motion‐sampling plots as a framework for predicting motion artifacts.

**Methods:**

We evaluated a range of motion‐sampling combinations across three sampling trajectories (Cartesian, stack‐of‐stars, kooshball) in both simulation and in vivo. Experiments included shifting motion states in k‐space, changing the direction of motion with regards to the sampling, and varying the magnitude of motion. In vivo experiments were conducted on healthy volunteers mimicking patient motion while wearing a real‐time pose‐tracking device. Motion‐sampling plots were used to map motion states directly onto k‐space and assess their relationship to artifact appearance.

**Results:**

Nine categories of motion artifacts were identified. The severity and nature of artifacts were found to depend heavily on the k‐space distribution of motion states. Motion‐sampling plots were seen to work as guides in predicting artifact appearance. In vivo findings supported simulation results. Artifacts were especially pronounced when motion discontinuities occurred near the center of k‐space or aligned with slow phase‐encoding directions.

**Conclusion:**

Motion‐sampling plots offer an effective way to visualize and interpret motion artifacts in 3D MRI, providing insight beyond traditional motion‐time plots. This framework enables systematic evaluation of motion robustness and can guide the development and validation of motion correction techniques. We propose practical recommendations for motion experiment design to improve reproducibility and benchmarking in MRI research.

## 1 Introduction

### 1.1 Objectives

Motion is a severe problem for MRI robustness, particularly for multi shot sequences that have a large temporal footprint, such as 3D sequences. In these sequences, any motion during the scan duration leads to inconsistent data in different parts of k‐space. This can cause artifacts in the resulting images, which can produce reduced clinical utility.

It is well known that the effect of motion on image quality depends heavily on which part of k‐space is sampled when the pose changes occur [1], and methods exist that incoherently sample k‐space to purposefully spread the resulting artifacts spatially and enable self‐navigating reconstruction methods (e.g., DISORDER [2] and golden angle [3] sampling). Repeated sampling of the center of k‐space is another tool to reduce the visual degradation due to motion, for example using radial [4, 5] or PROPELLER [6] sampling to average out the motion‐induced phase errors at the center of k‐space. So, although many methods rely on changing sampling to address the motion problem, the characterization of motion‐induced artifacts in the literature does often not go beyond broad statements such as “The aliasing artefacts manifest as ‘ghosting’ in 2DFT and EPI trajectories, and ‘streaking’ or ‘swirling’ in radial and spiral trajectories” [7]. A clear mapping of motion‐sampling interactions and the visual appearance of motion artifacts has not been studied, especially regarding 3D trajectories. Additionally, common statements such as “radial sampling is more robust to motion than Cartesian sampling” [8] can also be misleading as, again, the distribution of motion states in k‐space matters, and the same motion track does not necessarily produce less disruptive artifacts with radial sampling than it would with Cartesian even if it can be true on average. In this work, we formally assess and categorize a multitude of artifact types that arise because of rigid motion during the sampling of 3D MRI data, focusing on three common sampling methods: Cartesian sampling, radial stack‐of‐stars sampling, and radial kooshball sampling. Furthermore, we propose the use of *motion‐sampling plots* as an alternative to traditional *motion‐time plots* when discussing motion in MRI, and show how motion‐sampling plots can be used to intuitively predict the effect that motion will have on the resulting images.

*Motion‐sampling plots* were developed as part of this project to communicate which parts of k‐space were acquired in what pose, a feature that is missing from traditional *motion‐time plots*, where a subject's motion states are only reported with regards to time. We have previously demonstrated that different motion patterns (e.g., a small number of instantaneous pose changes and continuous fast periodic pose changes) that lead to similar motion‐sampling plots result in similar looking artifacts regardless of motion‐time profile [9].

This article builds upon that work, formalizing the use of motion‐sampling plots for communicating the effect of motion on image quality. Through experiments we demonstrate how simple motion patterns can have vastly different effects on image quality depending on the:

1. k‐space locations of motion states
2. Direction of pose‐change with regards to the directional ordering of k‐space sampling
3. Magnitude of pose change
4. Whether the motion induces secondary motion effects such as B_0_ inhomogeneities.

Finally, we propose a checklist of motion‐sampling interactions to consider in motion experiments for novel motion correction methods, to allow researchers to demonstrate method robustness to a broad range of motion conditions.

### 1.2 Background on Motion in Neuroimaging

For neuroimaging purposes, and for the purposes of this article, any motion of the brain is approximated as rigid, as the brain moves as a unit with the rigid skull. Rigid motion can be modeled with six parameters, translations along the X (left–right), Y (anterior–posterior), and Z (superior–inferior) axis, and three rotations (pitch, roll, and yaw, rotating around the physical X, Y, and Z axis, respectively). However, when a patient or volunteer is imaged while lying on their back, the majority of motion is rolling the back of their head against the cushion [10], which means that focusing on rotational motion often is sufficient to describe the motion states. Padding is frequently used to minimize left–right rotations (yaw) and sideways up‐down rotations of the head (roll), but there is commonly nothing stopping the patient/volunteer from performing nodding rotations (chin moving up‐and‐down, pitch), and this is thus generally the most common direction of motion [10, 11]. The magnitude of a translational motion measurement is dependent on the arbitrarily chosen center of rotation, which is not comparable between scans and sites. In addition, translations often happen with correlated rotation as long as the head is resting against the cushion [12]. Therefore this paper will focus solely on rotational motion as a method to reduce the dimensionality of the displayed motion‐sampling plots. Other methods of describing motion state using fewer than six parameters exist, for example, using the maximum or mean displacement over a sphere or of the brain itself [13, 14, 15, 16], but as these lose information related to the direction of motion they are not appropriate for our experiments.

In motion experiments, volunteers are often asked to perform simple movement patterns such as quickly moving from one pose to another during the scan, referred to as *instantaneous motion*. They can also perform different kinds of continuous motion, such as *slow drifts* from one pose to another, or periodic movement patterns, where different poses are repeated periodically during the scan. In reality, patient/volunteer motion is often a combination of slow continuous motion due to pillow deformation or relaxing of neck muscles during the scan, and instantaneous motion interleaved with periods of very little motion [15]. The magnitude and occurrence of these movement patterns vary greatly depending on the population being imaged. Healthy volunteers often move very little, whereas patient populations can move a lot during the scans (pediatric patients commonly move over 13° peak‐to‐peak and spend over 11% of the total scan time in motion [10]). Young pediatric patients also tend to move more than older ones [15]. In this article motion magnitudes up to ±10° are studied and both simple motion patterns and real pediatric patient motion patterns are explored.

## 2 Methods

To assess motion and k‐space sampling interactions, a series of experiments was designed covering both simulations and in vivo acquisitions. In the simulations, only rigid motion with a single homogeneous receive channel was simulated, whereas in the in vivo experiments secondary motion induced phase errors, effects from motion relative to heterogeneities in the transmit and receive fields, and partially non‐rigid motion (e.g., facial and tongue movement or blood and CSF flow), were inevitably included. As slab‐selective excitation was used in the experiments, some effects of out‐of‐slab motion could also be present.

**FIGURE 1:**
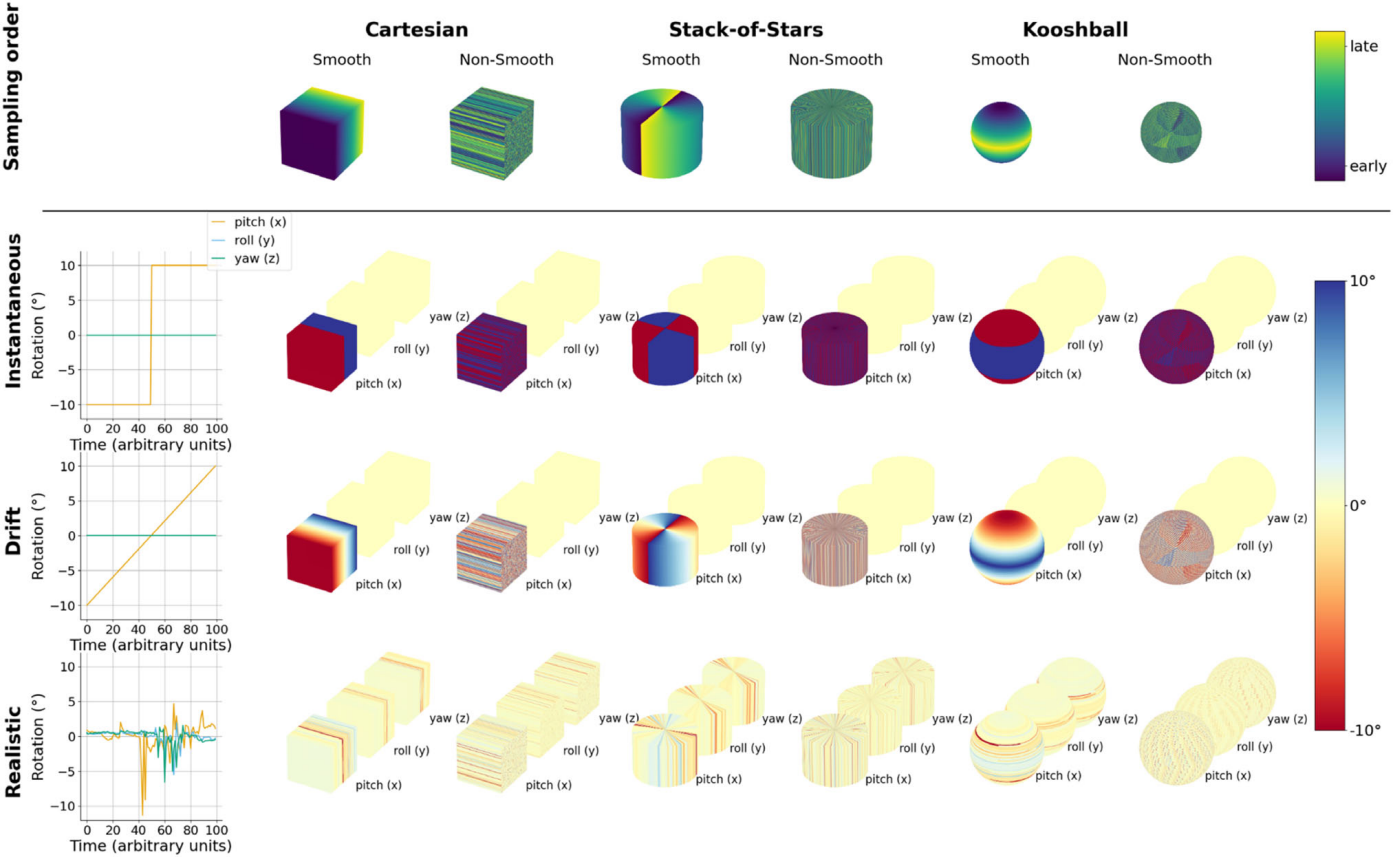
The different motion‐sampling combinations explored in depth in this paper. The top row shows the 6 different sampling patterns used (3 trajectories with two orderings each), and the bottom three rows show the motion‐sampling plots, visualizing how the different motion states are distributed in k‐space using the corresponding sampling patterns. The leftmost column displays traditional motion‐time plots that show how the subject's pose changed over time.

We have limited the scope of this study to 18 different motion‐sampling variations across two sampling dimensions and one motion dimension as shown in Figure 1 and described below:

1. Sampling (trajectory):
  - Cartesian
  - Stack‐of‐Stars
  - Kooshball
2. Sampling (ordering):
  - Smooth
  - Non‐smooth
3. Motion:
  - Instantaneous
  - Drift
  - Realistic

Variations of these that are described in some of the experiments below, for example, rotations around a different axis, varying the magnitude of motion, and temporal shifts of the motion traces, are not shown in Figure 1. All motion‐sampling plots use the average position throughout the scan as reference for the motion parameters (0 mm displacement and 0° rotation).

### 2.1 Simulations

All simulations were performed in Python using the SigPy toolbox for MRI modeling [17].

Motion was simulated by rigidly transforming each frame of a 130 × 130 × 130 voxel 3D Shepp‐Logan Phantom with 100 frames. *Instantaneous* motion was simulated by an instant change from one frame to another, whereas *drift* was simulated by linearly interpolating the rotation angles between all frames. For these cases, rotations were “pure rotations” around the center of the image. The realistic motion pattern included both rotations and translations of the digital phantom, where the translation parameters both moved the center of rotation and included “real” translations. Spin history effects were ignored assuming a non‐selective excitation.

For the 3D *Cartesian* experiments, data were fully sampled on a grid with isotropic resolution and isotropic field‐of‐view (FOV). For *smooth* sampling, the k_y_‐k_z_ coordinates were sampled in a nested loop, where the outer loop selects a ky value (from low to high), making it the slow phase encoding direction, and the inner loop iterates over all possible k_z_ values from low to high, making it the fast phase encoding direction. Throughout this article directions labeled X, Y, and Z correspond to the Cartesian readout, slow phase encoding, and fast phase encoding direction respectively. Motion in physical directions are referred to as pitch, roll and yaw. For *Non‐smooth* sampling, the order of k_y_‐k_z_ samples was randomized using a fixed seed for repeatability.

In the *stack‐of‐stars* sampling experiments, full‐spoke stack‐of‐stars sampling was used, with the k_z_‐direction fully sampled in a Cartesian fashion, and the k_x_‐k_y_ plane sampled using radial spokes. Every k_z_ coordinate at a given angle was sampled before moving to the next spoke. The number of spokes was 205, which was chosen to be at the theoretical Nyquist limit (*N* × 𝛑/2, where *N* = 130 is the number of phase encoding lines needed for Cartesian sampling with the same resolution and FOV). The spokes were extended in k‐space by a factor of √2 to reach the corner of the corresponding Cartesian grid. For the *smooth* sampling case, uniformly spaced spokes with a rotation 0° ≤ θ < 180° were used. Tiny golden angles [18] (tiny‐level = 3) were used for the *non‐smooth* sampling to ensure quasi‐random ordering.

In the *kooshball* sampling experiments, full‐spoke sampling was again used, where the theoretical Nyquist limit was used to determine the number of spokes (*N* ^2^ × 𝛑/2). The spokes were extended by a factor of √3 to reach the matrix corners. In total 26 547 spokes were used. In the *smooth* case, a single Archimedean spiral [19] was used to distribute the spokes on a half‐sphere, whereas in the *non‐smooth* case, a level 3 tiny golden means distribution [20] was used for quasi‐random sampling.

### 2.2 In Vivo Acquisition

All in vivo data were acquired on healthy volunteers (5 subjects, one female) in accordance with ethical approval issued by the Swedish Ethical Review Authority on a single GE Healthcare 3 T Signa Premier scanner with a 48 channel head coil. A 3D spoiled gradient echo sequence was used with TE = 2 ms, TR = 8 ms, and flip angle = 15°. For the in vivo experiments, the FOV was 24 × 24 × 24 cm^3^ and the matrix size 160 × 160 × 160 (1.5 mm^3^ isotropic resolution). The other sampling details were matched to the simulation experiments.

During the in vivo scans, the volunteers were wearing a Wireless Radiofrequency Triggered Acquisition Device (WRAD [21]) on their forehead, which measures its own position in space every TR during the sequence. Instead of using the WRAD for prospective motion correction, which it was designed for, it was simply measuring its own position. The current position (rotation parameters) was displayed on a monitor as a green “X” for the volunteer in real‐time along with a time synchronized pose target (red “X”) for the volunteer to follow (software available on https://gitlab.com/neuromr_karolinska/mocovisualisation). By changing the target position on the monitor based on the target rotations, the volunteer could accurately follow the target and perform the motion required for each experiment to mimic the simulated motion paths (See Figure S1 for the resulting accuracy with which the volunteers were able to mimic patient motion).

*Instantaneous* and *drifting* motion was done over ±5.0° rotations around the x‐axis (nodding motion). *Realistic* motion patterns were acquired by asking the volunteers to follow patient recordings from the https://brainmrimotion.org database. We used the motion tracks of five unsedated pediatric patients (3 male, 2 female, ages 4–10) from a previous study [10] (further details about the subjects, including how to find the recording in the database can be found in the Supporting Information). When the target motion data from the database had a different length/number of readouts than the acquired sequence (25 600 for Cartesian, 40 213 for kooshball, and 40 320 for stack‐of‐stars), it was stretched using nearest‐neighbor interpolation to cover the whole sequence, so that all sequences covered the same motion states, but moving through them with 44%–70% of the original speed. Despite being slightly slower than the patient motion, the motion still seemed natural as the large movements often were fast instantaneous movements with long periods of relative stillness in between (Figure S1).

### 2.3 Experiments

#### 2.3.1 Characterization of Motion Artifacts

All 18 motion‐sampling combinations shown in Figure 1 were simulated for varying motion magnitude (small (±1.5°), medium (±5.0°), and large (±10.0°) motion [22]) and motion directions (pitch, roll, yaw) except the realistic motion which was only done using the same magnitude and motion as measured in the patient. The 18 motion‐sampling combinations were also executed in vivo by a single volunteer at a single magnitude (±5.0°) and direction (pitch). The simulated images were first analyzed in detail by one reviewer (SSc) using visual inspection and were described using qualitative features of the resulting images. The images were examined in all three planes and at different slice positions. This was done by visualizing the results using a purpose‐built dashboard that has been shared in the repository accompanying this paper.

After categorizing the expected artifacts from the simulation experiments, images from the volunteer were compared with the simulation results by the same reviewer. The presence of motion artifacts similar to those in the simulation were assessed visually. The findings from the in vivo experiments were then compared with the simulations again, to ensure no findings from the in vivo experiments had been missed in the simulation data assessment and vice versa. From these observations, a list of artifact descriptions and their correspondence to features in the motion‐sampling plots was produced.

#### 2.3.2 Effect of k‐Space Location of Motion State Discontinuities

**FIGURE 2:**
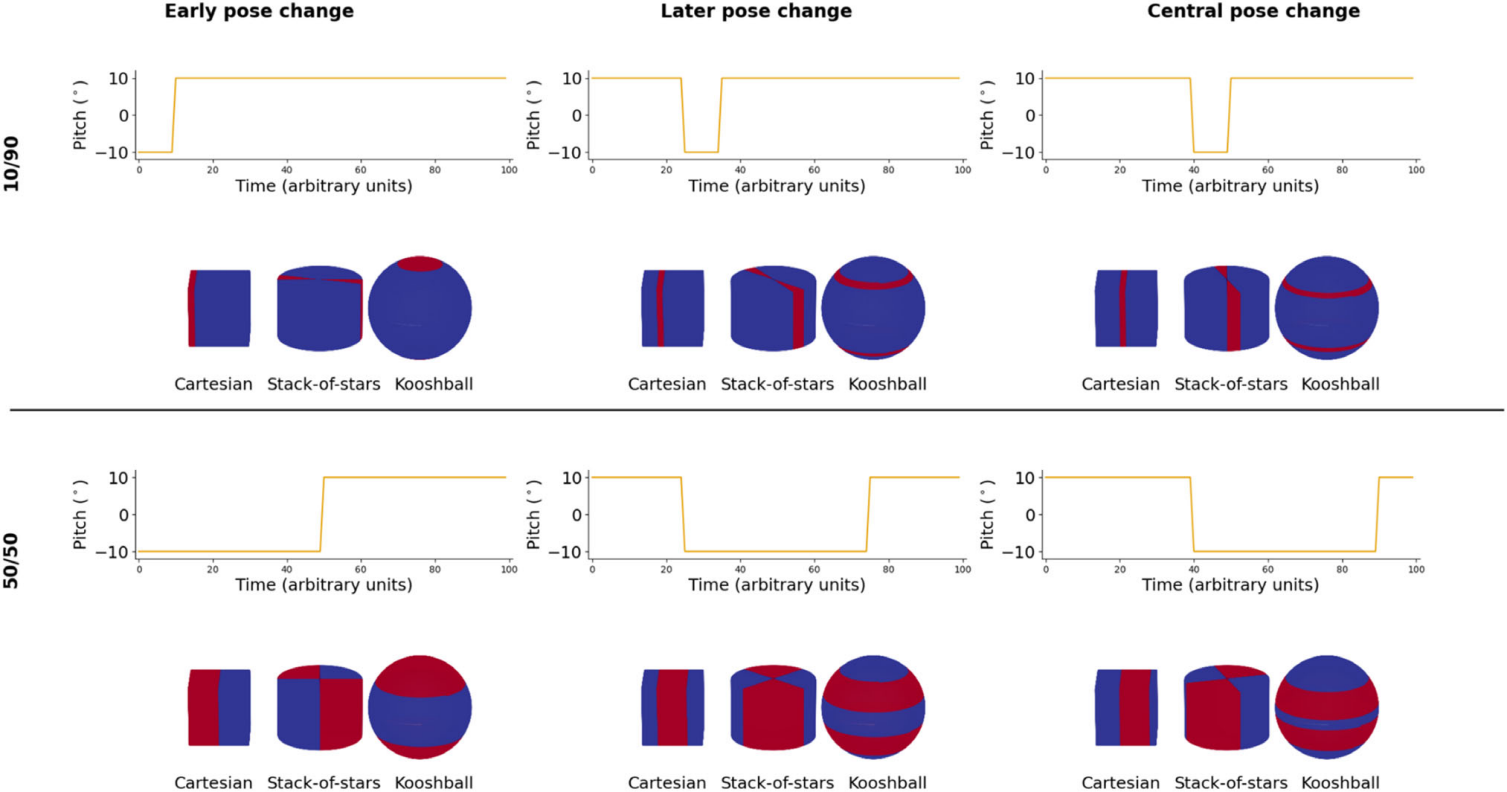
In this experiment there are two poses; negative and positive 10° rotation around the x‐axis. In the standard motion‐time plots the states are shifted along the time axis, which in combination with smooth sampling moves the states in k‐space, which can be seen in the corresponding motion‐sampling plots. The negative state is shown in red in the motion‐sampling plots (note that these plots have not been normalized to average position and show absolute position instead for clarity). Each column shows a different start time of the negative pose and the two rows show the experimental conditions with different amounts of the scan spent in each state.

Additional motion‐sampling effects were explored in simulation by moving the motion‐state discontinuities in k‐space while keeping the number of readouts in each state constant. This was done for the instantaneous motion case with smooth sampling. Temporal shifts were introduced such that the position of the discontinuity was shifted in k‐space. The negative rotation state was started at the beginning of the scan, 25% into the scan, or 40% into the scan. Additionally, the amount of time spent in each motion state was either split 10%/90% or 50%/50% (Figure 2). This experiment was performed only in simulation and only rotation around x with large motion (±10.0°).

#### 2.3.3 Effect of Direction of Motion

In simulation, directional experiments were done on the *drift* and *instantaneous* motion cases by applying the rotation on either the x, y, or z‐axis while keeping the sampling ordering unchanged as the direction of motion in combination with direction of sampling has previously been shown to affect the artifact types with Cartesian sampling in 2D [1, 23]. The images were viewed in different planes to compare the effect.

In the realistic motion experiments described below, the effect of direction was further explored by changing the acquisition direction from axial to sagittal in two subjects.

#### 2.3.4 Effect of Motion Magnitude

For the *drifting* and *instantaneous* motion cases, small (±1.5°), medium (±5.0°), and large (±10.0°) motion were simulated. The drifting motion around the x‐axis case was also performed at the same magnitude levels in vivo to compare loss of image quality when secondary effects such as motion induced B_0_ inhomogeneity are present.

#### 2.3.5 Motion‐Sampling Plots and Realistic Motion

**FIGURE 3:**
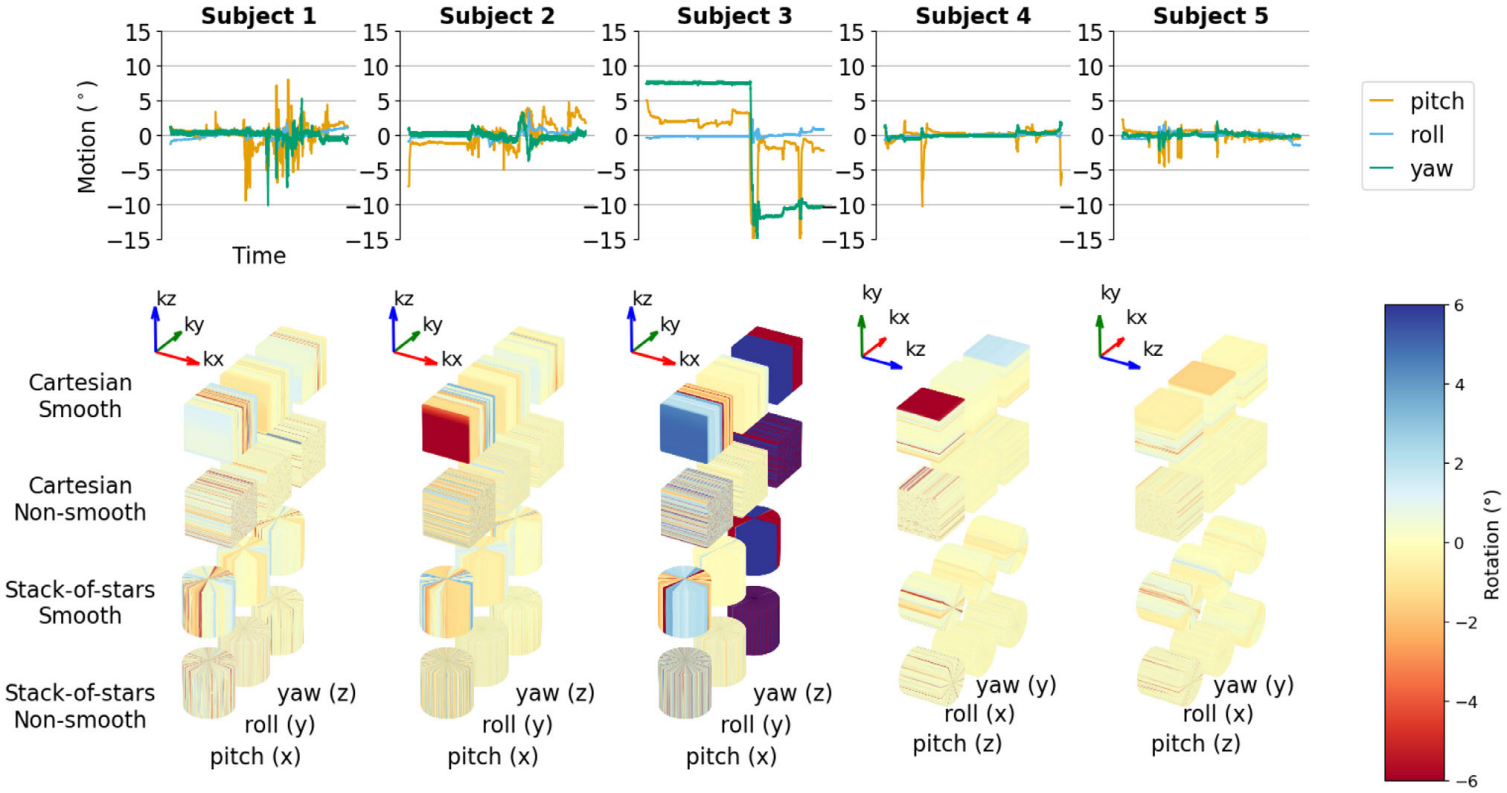
The five motion tracks (left to right) used to assess realistic motion in combination with different sampling patterns. The motion‐sampling plots show how the motion states get distributed in k‐space for the four sampling cases tested in vivo.

Five volunteers performed realistic motion tracks mimicking pediatric patient motion in combination with Cartesian and stack‐of‐stars sampling with both smooth and non‐smooth ordering (Figure 3). Subjects 1–3 were acquired axially, with the readout in the left–right direction in the Cartesian case and the axial plane corresponding to the radial plane in stack‐of‐stars. Subjects 4–5 were acquired sagittally with the readout in the anterior–posterior direction in the Cartesian case, and the sagittal plane acquired radially for stack‐of‐stars. These images were then assessed for motion artifacts in the previously identified artifact categories by five reviewers (AvN, EA, ON, HR, SSk), who are MR physics experts and familiar with the signal processing basis for motion artifacts. The reviewers were blinded to the sampling condition. For each motion‐sampling condition, and for the artifacts identified by at least two of the reviewers, an attempt was made to explain them using their corresponding motion‐sampling plot. This allowed for assessment of whether the previously identified categories sufficiently describe the motion artifacts and whether the motion‐sampling plots can give a heuristic way of explaining how the artifacts are likely to be manifested.

## 3 Results

### 3.1 Describing and Categorizing Artifacts

#### 3.1.1 Artifact Descriptions

**FIGURE 4:**
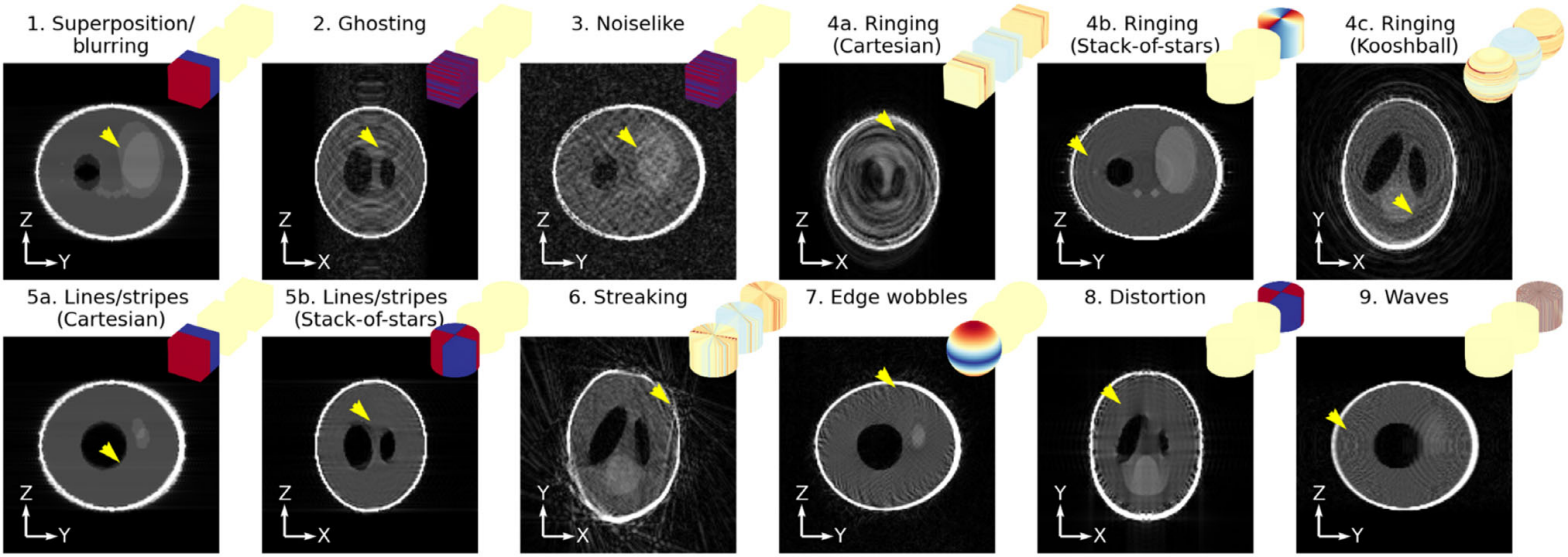
Examples of the identified artifact types in simulated data along with their motion‐sampling plots. Note that 1 and 5a, as well as 2 and 3 have the same motion sampling plot, but demonstrate different artifacts arising in a different slice/direction of the imaged volume. Yellow arrowheads point out the artifacts.

**FIGURE 5:**
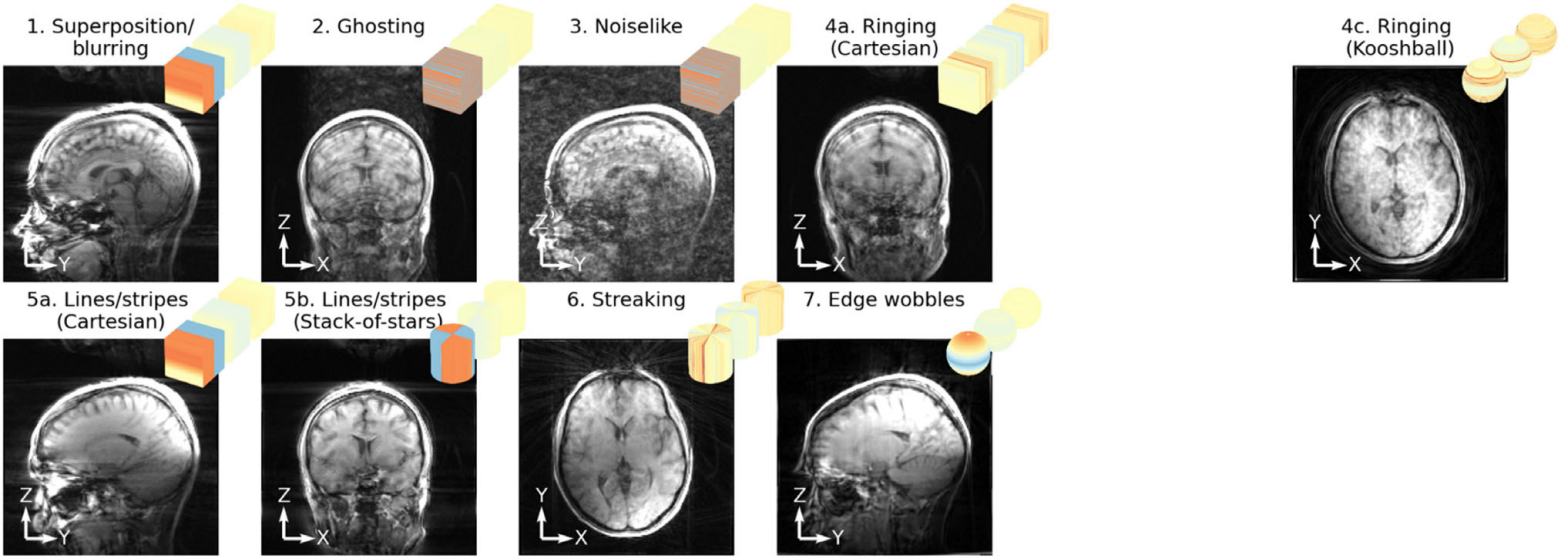
Examples of the identified artifact types in in vivo data along with their motion‐sampling plots.

In total, nine different categories of artifacts were identified in the 18 motion‐sampling combinations, of which most were observed in vivo as well. The ones that weren't identified in vivo were the ones where stack‐of‐stars sampling was used in combination with motion around the z‐axis, which was not performed in vivo. Examples of each are shown in Figure 4 (simulation) and Figure 5 (in vivo) and the categories are described below along with the features in motion‐sampling plots that predict them:

1. Superposition/blurring—This is the most common effect and was visible in reconstructions where the area near the center of k‐space was sampled in multiple motion states (all motion patterns with kooshball, and stack‐of‐stars, as well as instantaneous pose change around the center with Cartesian sampling).
2. Ghosting—This is a typical Cartesian artifact that was visible when the motion states were spread in a stripy pattern in k‐space. Ghosting occurs with any motion in combination with non‐smooth sampling order, as well as smooth sampling order in combination with a quickly changing pose of the realistic motion pattern. This effect is highly directional, depending on which directions in the motion‐sampling plot contain the stripy pattern.
3. Noiselike—This effect happens when motion states are randomly distributed in k‐space, which was observed when sampling with random ordering with either Cartesian, stack‐of‐stars, or kooshball trajectories. For Cartesian sampling, this was only in the plane containing both phase encoding directions.
4. Ringing
  - Cartesian—This effect appears when there are areas of through‐plane rotations (e.g., pitching motion for a coronal view) with a discontinuity in k‐space parallel to the viewing plane.
  - Stack‐of‐stars—Similarly, a discontinuity in motion states causes this effect in the plane containing the Cartesian direction of stack‐of‐stars sampling. This effect was mild and only observed in the simulated data potentially due to other effects being dominant in vivo and through‐z rotations not being performed in vivo.
  - Kooshball—Ring‐like artifacts could also be seen in kooshball sampling when there were consistent conical rings of a motion state in k‐space. This happened with smooth sampling in conjunction with the realistic motion.
5. Lines/stripes
  - Cartesian—This effect appears at edges when there is a discontinuity in motion state in the middle of the studied k‐space plane (e.g., *instantaneous* pose change + smooth sampling). The stripes appear in a direction perpendicular to the discontinuity.
  - Stack‐of‐stars—Similar to the Cartesian case, this effect appears when there is a discontinuity through the center of k‐space in the plane containing the Cartesian direction of stack‐of‐stars. This discontinuity is present both with instantaneous pose change and drifting motion with smooth sampling as the radial spokes only swept over 180° of the circle producing a discontinuity when the starting pose differed from the end pose.
6. Streaking—This is a typical radial effect when wedges of k‐space are in a different motion state than the rest of k‐space. This appeared both with stack‐of‐stars and kooshball sampling. The direction of the streak in the image is perpendicular to the motion corrupted wedge in k‐space and appears in areas of the image where the motion is the largest, often at edges close to the edge of the FOV.
7. Edge wobbles—Smooth motion variation across k‐space (drift with smooth sampling) can cause edges to display small “wobbles” when the high frequency data is inconsistent with the low‐frequency data. This effect was observed with both Cartesian and kooshball trajectories.
8. Distortion—This effect was seen with stack‐of‐stars and kooshball sampling when different head rotations corresponded to consistent spoke directions, such that the shape of the phantom (ellipsoid) got distorted. This effect was very clear in simulation, where the object had a very smooth and well‐defined shape but not noticeable in vivo potentially due to the direction of motion as well as smaller motion magnitude.
9. Waves—The final effect was only seen in simulation and was a radial effect (both in stack‐of‐stars and kooshball imaging with drifting and instantaneous motion) consisting of waves bending in the opposite direction to the edges in the image. This is likely a secondary effect of purely rotational motion in combination with radial sampling and could be obscured by other effects in vivo.

### 3.2 K‐Space Location of Discontinuity

**FIGURE 6:**
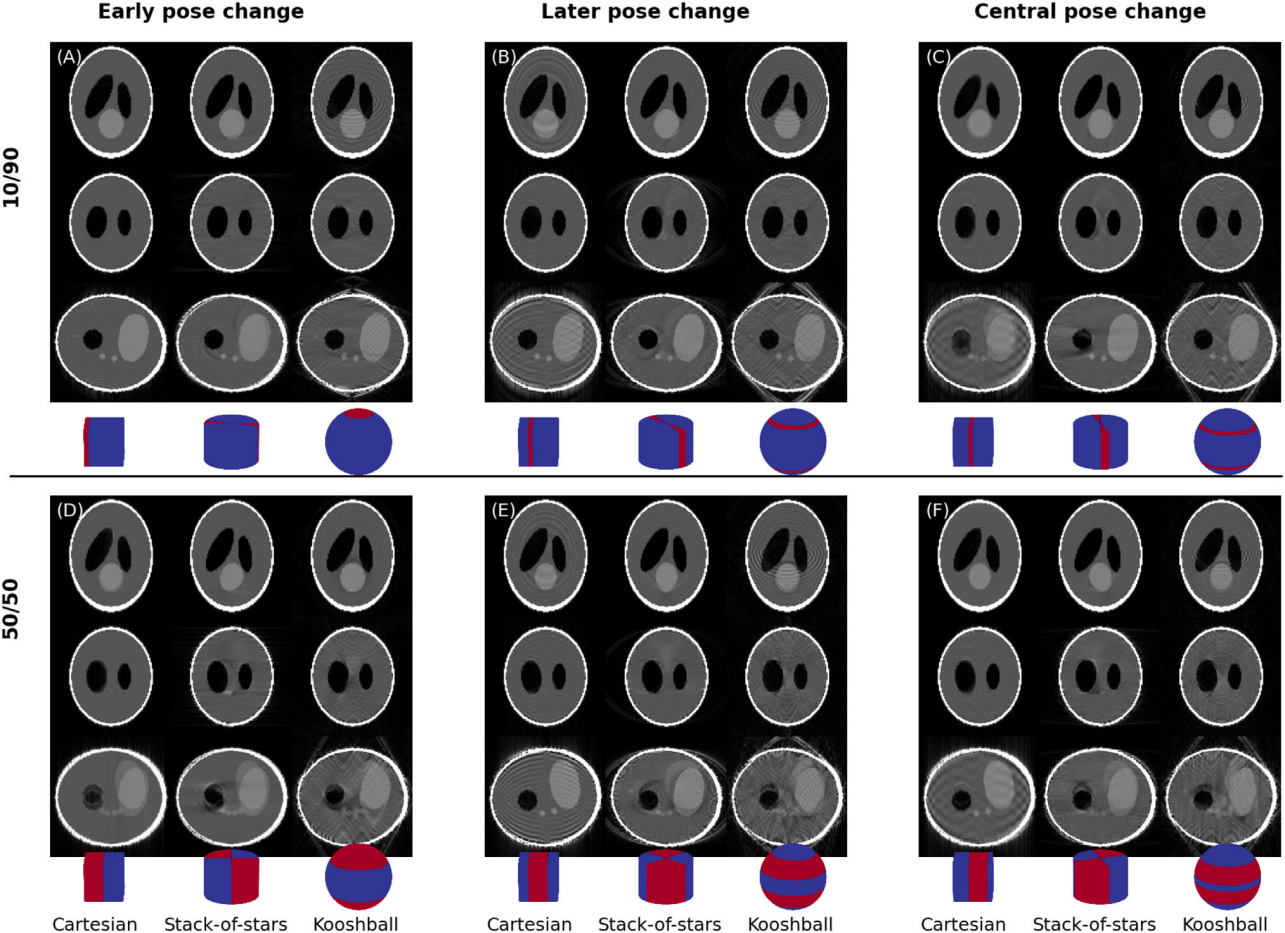
The top row (A–C) shows the effect of sampling 90% of the data in one pose (+10° pitch) and 10% in another (−10° pitch), whereas the bottom row (D–F) shows the effect of a 50/50 split. The columns depict a shift in time when the pose change happens, and thus, which part of k‐space gets corrupted, as shown in the overlaid partial motion sampling plots (positive pose in blue, and negative in red).

Disrupting the first 10% (Figure 6A) has a minimal effect on the image for Cartesian sampling, as only a small amount of the high‐frequency components of k‐space, which carry very little energy, are affected. With stack‐of‐stars or kooshball sampling, the effect is more pronounced as each spoke carries equal energy and thus a data inconsistency produces more severe artifacts. As the timing of the pose update changes (Figure 6B,C), the artifact level increases for Cartesian sampling, whereas for the radial methods, the direction and appearance of artifacts change in the different planes, but their severity remains constant.

When 50% of the samples are motion‐corrupted (Figure 6D–F), similar effects are seen, where motion‐artifact appearance changes significantly depending on when the pose changes occur. Again, for the radial cases, the timing strongly affects the directionality of artifact and highlights the need to view and assess artifacts in multiple planes.

### 3.3 Motion and Sampling Directions

**FIGURE 7:**
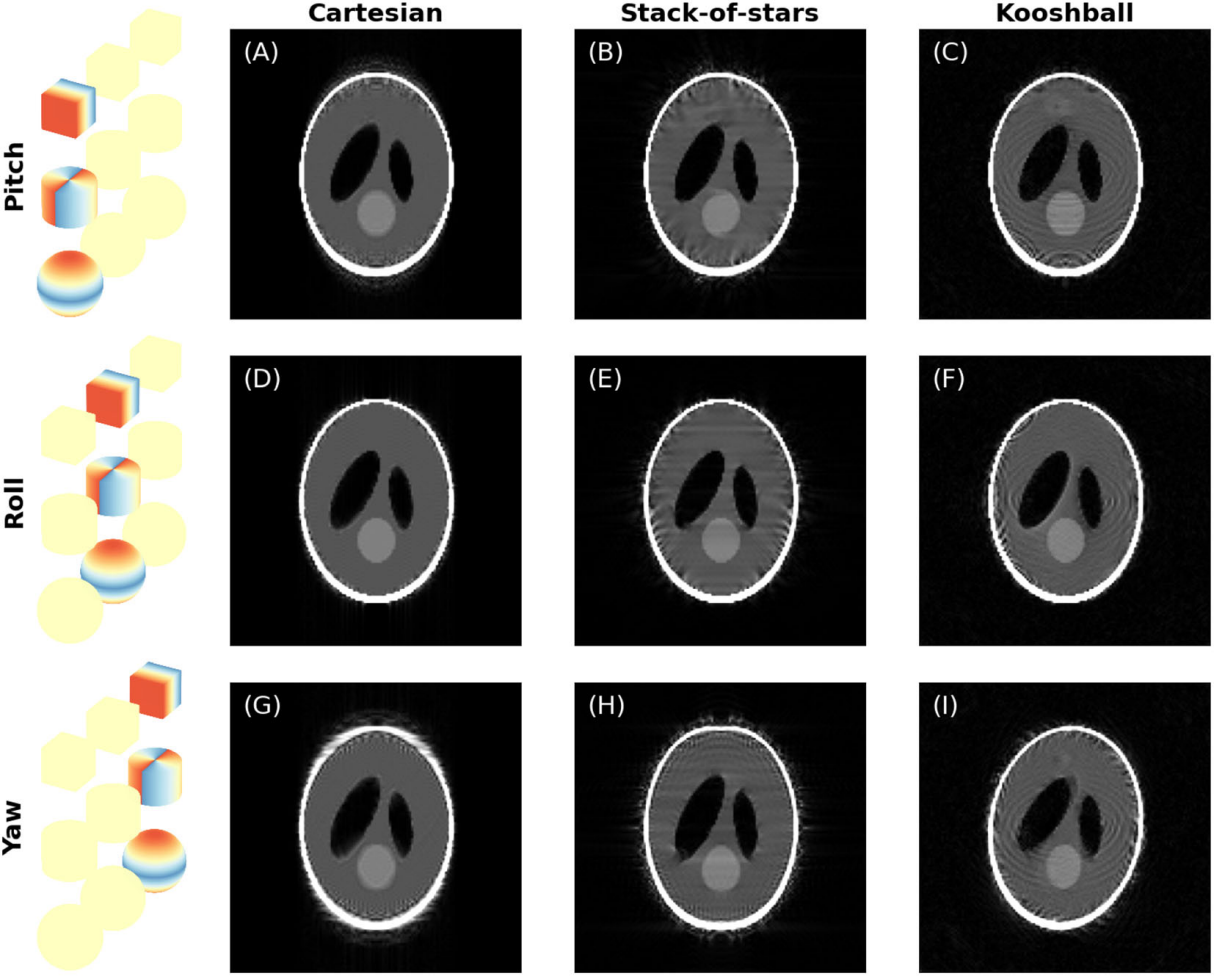
The effect of motion around different axes (pitch, roll, yaw) shown in the three rows of this figure. The effect of motion is generally larger for in‐plane motion (G–I) than for through‐plane motion (A–F). In the Cartesian case (A, D, G) the artifacts appear along the slow phase encoding direction (up‐down in the image).

The appearance of artifacts was found to be heavily influenced by the direction of motion with regards to sampling directions. An example of this can be seen in Figure 7 for the example of drifting motion in combination with smooth sampling and the three different trajectories. One of the main observations on motion direction is that in‐plane motion (motion in the plane the image is viewed in) often causes more noticeable artifacts than through‐plane motion. However, the motion state distribution, especially discontinuities, regardless of the direction of motion seems to be a larger factor in driving motion artifact appearance.

The directional ordering of k‐space lines has a large effect on the types of artifacts visible in the different planes. In the smooth Cartesian case, the effect of motion is very different in the frequency encoding direction (k_x_), fast phase encoding direction (k_z_ in our case) and slow phase encoding directions (k_y_). In contrast, for the non‐smooth case, both phase encoding directions are equivalent. As a rule of thumb, it is worth considering that motion primarily affects the image in planes that include the slowest phase encoding direction, as every k‐space plane in it encompasses poses from the entire scan duration.

In the stack‐of‐stars case, we have a fixed Cartesian fast phase encoding direction (k_z_), but the radially sampled k_x_‐k_y_ plane is mostly rotationally invariant (except for the discontinuity direction, where, in the smooth case, the sampling starts and finishes). In this case, the Cartesian phase encoding direction is sampled faster than the radial planes, and thus motion effects in the k_x_‐k_y_ plane are likely to be more pronounced than effects along k_z_.

In the kooshball case, all sampling directions are equivalent in the sense that each readout line carries the same energy. However, with smooth sampling, the artifact appearance in the different planes vary as the effect of motion depends on which pole the trajectory is spiraling from (this effect is seen in Figure 6). With non‐smooth kooshball sampling all directions are equal.

### 3.4 Motion Magnitude

**FIGURE 8:**
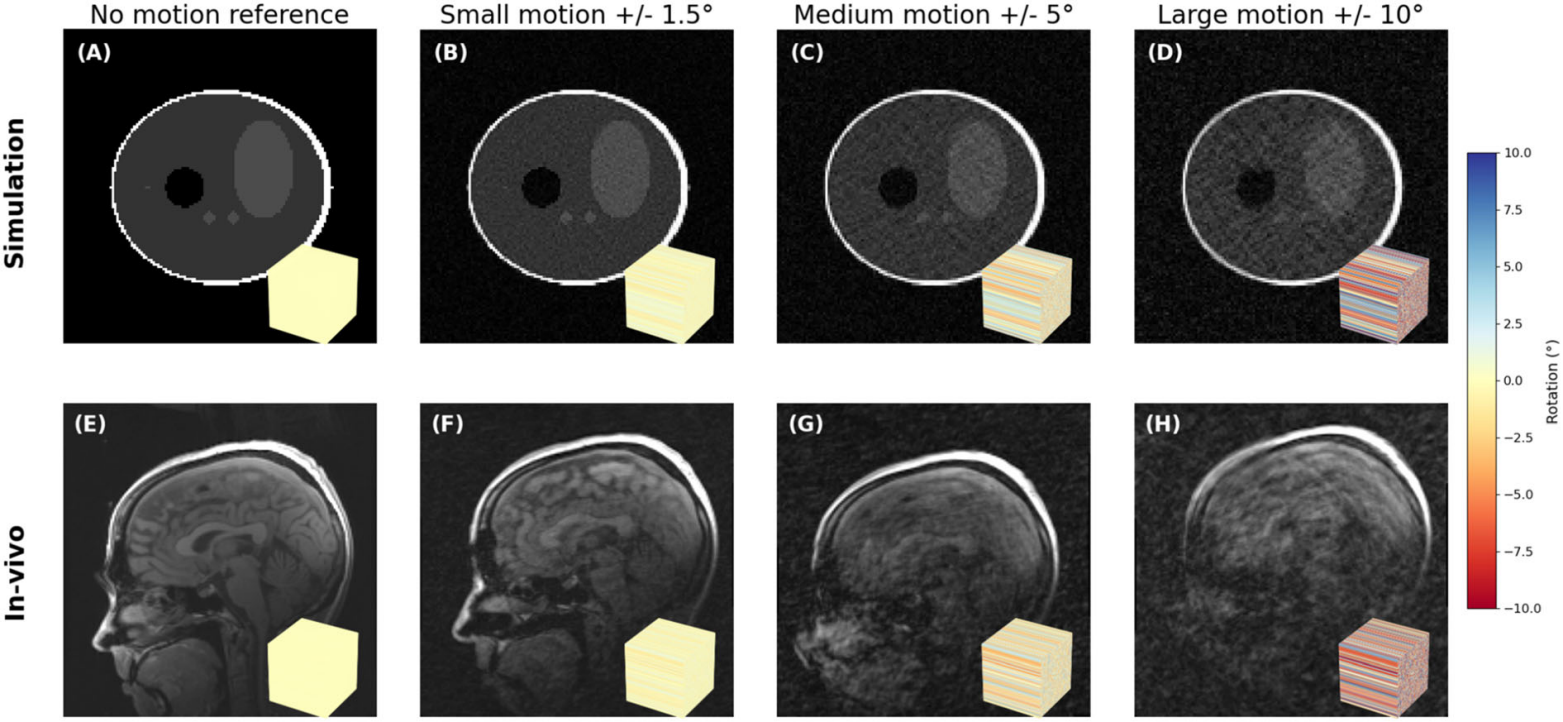
Simulation (A–D) and in vivo (E–H) appearance of increasing in‐plane motion (pitch) in combination with non‐smooth Cartesian sampling. In the in vivo case, the artifact severity, especially around the face and sinuses, is worse due to the secondary effects of motion. Even small motion can produce severe artifacts.

In simulation, the magnitude of the motion affects the degree of artifact in a straightforward way with larger amplitude increasing the severity of the artifact but not substantially changing its appearance, whereas in vivo larger motion also induces further artifacts from secondary motion imperfections (through coil motion, induced B_0_ inhomogeneity, etc.). Figure 8 shows the effect of the three different magnitudes of *drifting* motion with *non‐smooth Cartesian* sampling in simulation and in vivo as an example. Here, with increasing motion magnitude in simulation, the resulting image gets increasingly blurry and noisy, but the effect is more pronounced in vivo, especially around the neck and sinuses where the motion is largest and B_0_ induced, as well as non‐rigidity effects are most severe.

### 3.5 Artifacts in Realistic Motion Data

**FIGURE 9:**
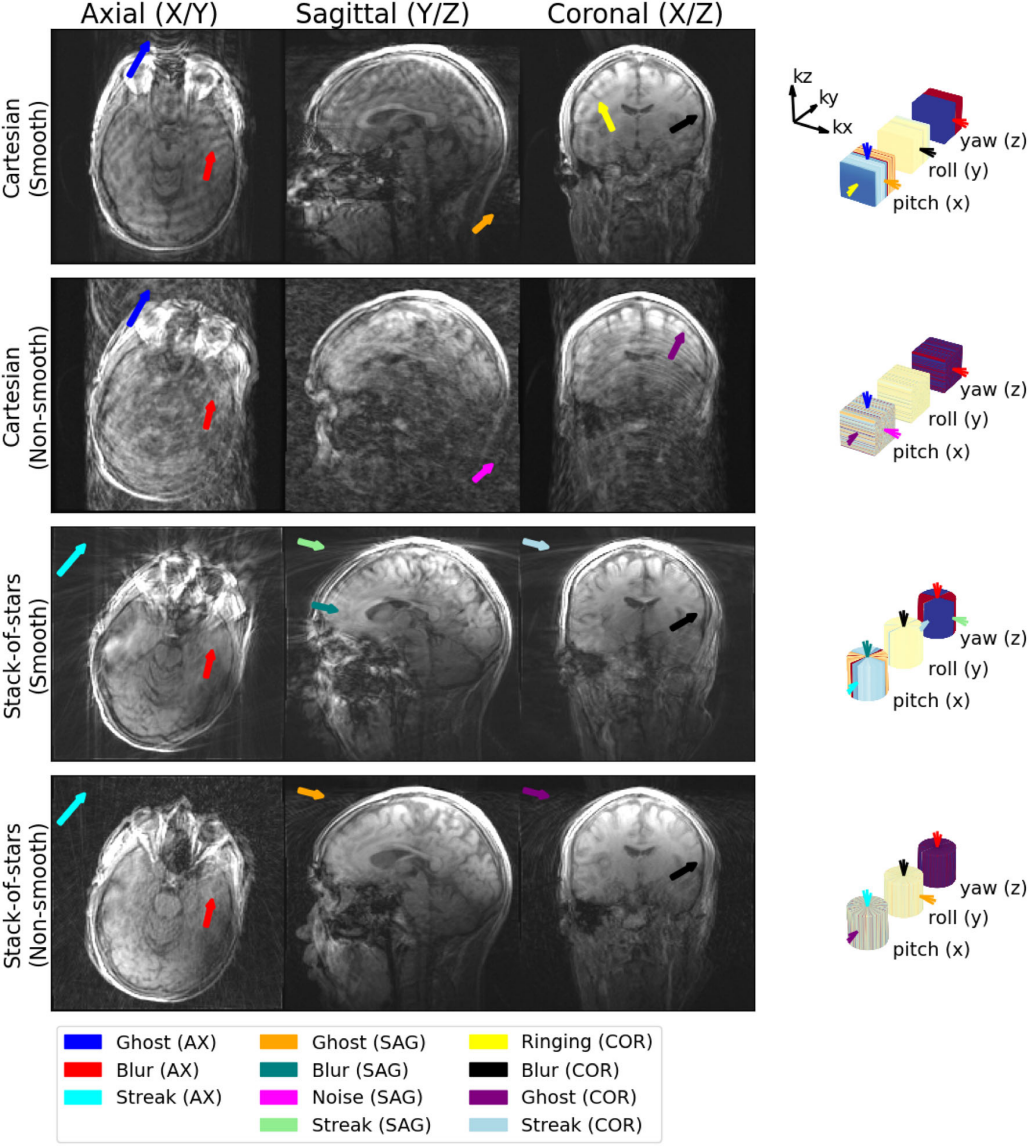
Artifacts identified by the blinded reviewers in the images acquired axially (x‐y plane is in the axial view) with extreme motion.

All subjects' motion led to severe motion‐artifacts, but their appearance and severity varied significantly between the different sampling strategies. At least two of the reviewers identified blurring in 19 of the 20 examined images. Ghosting was found in 9 out of the 10 images sampled with Cartesian sampling, and 3 of the 10 stack‐of‐stars sampled images. Streaking was identified in all 10 of the radially sampled images. Noiselike artifacts were identified in the plane containing both phase encoding directions in all subjects sampled with non‐smooth Cartesian sampling. Lines/stripes were identified in only 2 images, but potentially misclassified as streaking in one image (see Figure 9). The ringing artifact was found in the X–Z plane of all 5 images sampled with a smooth Cartesian trajectory as expected, but also identified in the X–Y or the Y–Z plane in 4 other images. Edge wobbles, distortions, and wavelike artifacts were not identified by at least two reviewers in any of the images. Other artifacts were not motion related, but rather identified as aliasing due to non‐optimal sampling direction in the sagittal cases and Gibbs ringing due to low resolution. The complete rating results are available in the supplemental material visually summarized in Figure S2.

**FIGURE 10:**
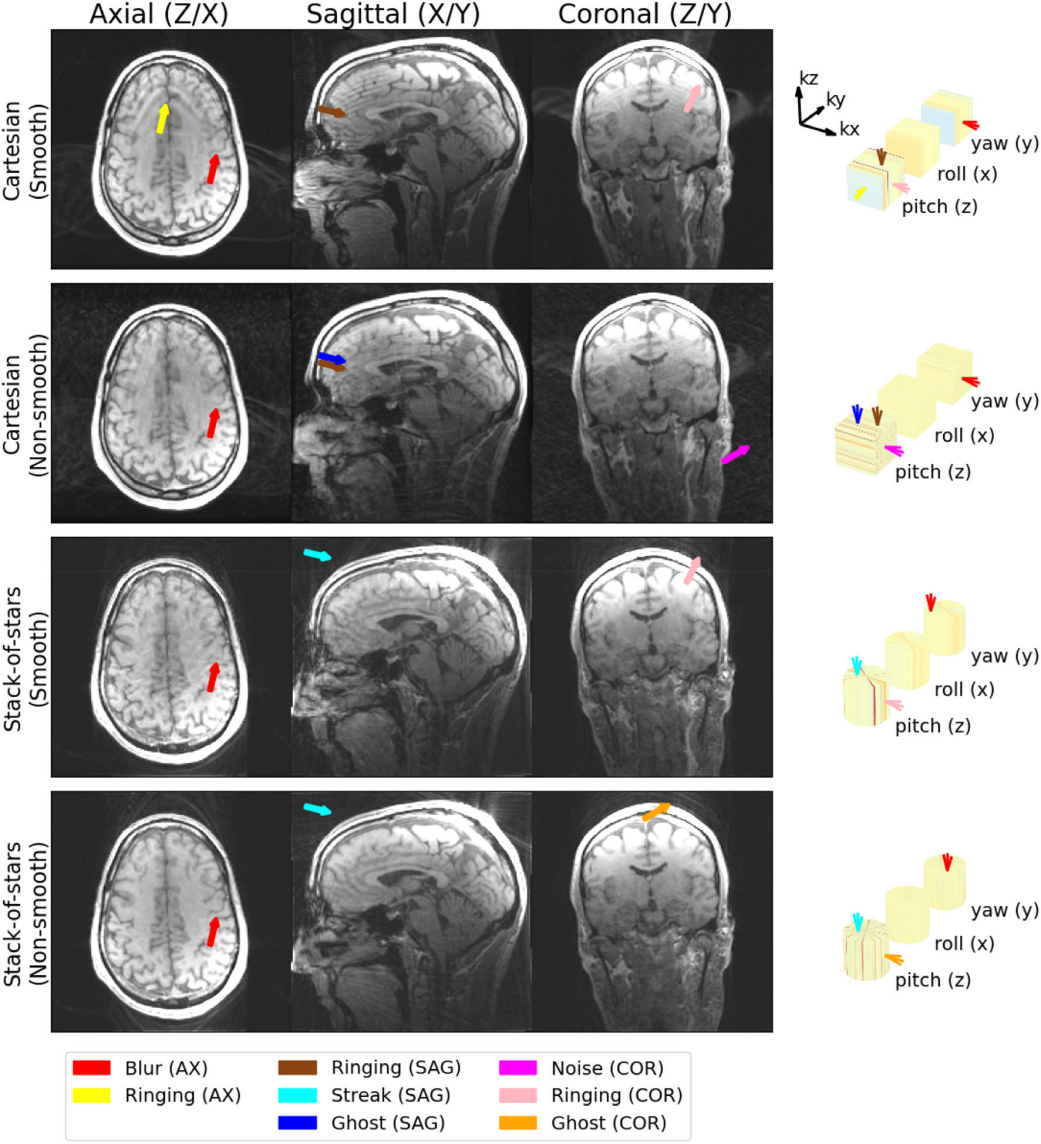
Artifacts identified by the blinded reviewers in the images acquired sagittally (x‐y plane is in the sagittal view) with moderate motion.

In Figures 9 and 10, we show two representative annotated subjects along with all their reviewer‐identified artifacts described using motion‐sampling plots; one with extreme motion acquired axially (Figure 9), and one with lower motion acquired sagittally (Figure 10). The complete list of subjects, along with their artifact scores and motion sampling plots, is shown in Figures S3–S7.

In the axial images we see ghosting (blue) due to stripy motion‐sampling plot appearance in the in kx‐ky plane, blurring (red) due to strong in‐plane motion (yaw) with two poses near the center of the motion‐sampling plot, and streaking (cyan) due to wedges in the kx‐ky plane of the SoS motion sampling plot with worse appearance in the smoothly ordered case as the wedges are more pronounced.

In the sagittal images we see ghosting (orange) for the same reason as in the axial case, but now only when the stripes are in the ky‐kz plane of the motion sampling plot, blurring (teal) due to multiple poses near the center of k‐space, mostly driven by pitching motion, noiselike (magenta) artifacts appear when motion states are randomly distributed in the ky‐kx plane, and streaking (pale green), which cannot be explained by wedges, but rather a misclassification of the lines/stripes due to a strong discontinuity in the center of the ky‐kz plane with SoS sampling.

In the coronal images we see ringing (yellow) due to through‐plane motion (pitch and yaw) with a discontinuity in k‐space parallel to the imaging plane (in the ky‐direction), there is also blurring (black) due to the extreme motion, but it would be worse with more in‐plane motion (roll) states near the center of k‐space, there is also ghosting (purple) due to stripes in the motion sampling plot (in the kx‐kz plane) and streaking (light blue), which also is a misclassification of lines/stripes as there is no radial sampling in the coronal plane.

In the axial images we see blurring (red) due to strong in‐plane motion (yaw) with multiple poses near the center of the motion‐sampling plot, and ringing (yellow) due to through‐plane motion (pitch and roll) with a discontinuity in k‐space parallel to the imaging plane (in the ky‐direction).

In the sagittal plane ringing (brown) was a misidentified artifact that was indeed ghosting (blue) due to stripy motion‐sampling plot appearance in the in kx‐ky plane in combination with fold‐over artifact due to the non‐ideal sampling direction. There was also streaking (cyan) due to wedges in the kx‐ky plane of the SoS motion sampling plot with worse appearance in the smoothly ordered case as the wedges are more pronounced.

In the coronal images we see ghosting (orange) for the same reason as in the sagittal case, but now only when the stripes are in the ky‐kz plane of the motion sampling plot, noiselike (magenta) artifacts appear when motion states are randomly distributed in the ky‐kx plane, and ringing (light pink) is a misclassification of ghosting due to the stripy ky‐kz plane in the motion sampling plot with SoS sampling.

## 4 Discussion

This study aimed to describe and categorize artifacts arising from various motion‐sampling interactions in 3D MRI, expanding on previous work in describing motion effects in MRI that focused on Cartesian 2D effects [1, 23]. This was done to provide good “rule‐of‐thumb” predictions of artifact appearance when specific features appear in 3D motion‐sampling plots. With a good model for expected artifact appearance, researchers can anticipate the type and severity of artifacts likely to occur under different motion‐sampling scenarios, enabling more informed choices in sampling strategy design and artifact reduction approaches. It also becomes easier to assess the efficacy of novel motion correction approaches. This predictive capability reduces reliance on exhaustive simulations [24], streamlines experimental planning, and supports more robust interpretation of image quality. Clinically, knowing which artifact type is most damaging for diagnosis, re‐acquisition schemes could be employed as soon as the motion‐sampling interaction predicts those artifacts, instead of waiting until the end of acquisition and reconstruction to discard the data.

In the experiments shown here, motion‐sampling plots showing only rotational motion were deemed sufficient with the assumption that the head mostly rotates around a fixed point and that translations without any rotational component are physiologically unlikely. However, should one be performing experiments where there is a lot of translational motion that is fully uncorrelated with the rotational motion (e.g., patients that often lift their head from the pillow), we recommend that the motion states are represented in a different way on the motion‐sampling plots for example, by showing translational motion plots alongside the rotational ones (in this case one have to be very careful with defining the origin of rotation and controlling for it so that the reported translations become physically meaningful) or by using other displacement metrics. An example of what this might look like has been included in Figure S8 for the interested reader. Further exploration into how motion states, described using various metrics, produce artifacts depending on their distribution in k‐space, could build upon efforts to automate reacquisition of motion corrupted k‐space lines [25, 26]. Similarly, one could imagine using metrics that show how each line in k‐space has been corrupted that incorporate spin history effects (e.g., having different motion‐sampling plots for different parts of the image), but this does make the artifact prediction problem much harder.

We identified nine different artifact categories, some with subcategories due to similar appearance but other features in the motion‐sampling plots that led to them. We note that the search space was limited to only 18 motion‐sampling combinations and their variations in terms of motion magnitude and direction. There are a near‐infinite number of possible motion trajectories and sampling approaches. As such, the categories presented here are not exhaustive but rather representative of a few common scenarios. While these categories provide a useful framework for understanding motion artifacts, future work should aim to expand the taxonomy to include more motion patterns, sampling strategies, and MRI sequences. Additionally, this work focused on the primary effects of motion with limited attention to secondary effects such as motion‐induced B_0_‐variation [27] and motion during signal readout. Translational motion was also mostly ignored due to its strong coupling with rotational motion in neuroimaging. Since similar image effects were seen in the in vivo experiments (with inevitable translations due to the performed rotations) as in simulations (with only rotation), this can be considered a reasonable simplification. These aspects could, however, be further explored in future studies, along with input from clinicians on which artifact types are acceptable to read through and which are more disruptive for diagnosis.

One key finding was, perhaps contrary to initial expectations, that stack‐of‐stars and kooshball trajectories did not always have more benign‐looking artifacts than Cartesian sampling. This was particularly well demonstrated in Figure 6A, where a small amount of motion at the edge of k‐space had a minimal effect in Cartesian sampling but a significant effect in radial sampling. This highlights the importance of showing where in k‐space motion occurred, especially for Cartesian sampling, where the energy carried by each k‐space line varies significantly, rather than only sharing summary metrics such as peak‐to‐peak displacement or root‐mean‐square displacement. Similarly, a motion‐time plot does not convey this information sufficiently. This observation can also be of high clinical importance as we learn more about how different patient groups move (e.g., the prominence of patients being startled in the beginning of the scan vs. restless toward the end of long scans), acquisitions can be better optimized to move the sensitive center of k‐space to a time when patients are most likely to be still.

A randomized (non‐smooth) sampling order (even ignoring effects from e.g., varying eddy currents) increases the probability of incoherent artifacts, which is exploited in for example, DISORDER. However, non‐smooth sampling can also enhance the appearance of artifacts by producing ghosting and noiselike artifacts from for example, drifting motion rather than the rather benign “edge wobble” effect when smoothness in k‐space is preserved. This, again, underlines the importance of having good heuristics for how motion‐sampling effects manifest in images, especially with Cartesian sampling. Furthermore, when considering ordering of lines in combination with parallel imaging or compressed sensing (not explored in this article), motion‐sampling plots could be employed to see the motion states in local neighborhoods of k‐space and, again, get a feel for how well or poorly advanced reconstruction methods would be able to “fill in the gaps” considering the range of motion states in the gap's neighborhood. Artifact prediction does, however, get harder when undersampling effects have to be considered too, as many undersampling artifacts have similar appearance to motion artifacts.

The direction of motion along with the direction of sampling are important aspects to consider when showing the effect of motion on images. In‐plane motion generally produces worse artifact appearance, so it is important to consider which direction the subject moved in with respect to the image plane shown in for example, a publication. In some cases, artifacts can be obscured simply by aligning the motion along a more forgiving plane. This also has clinical significance, as many radiologists have preferred directions to view different sequences/organs in even for isotropic 3D sequences, and motion artifacts can be made more benign by choosing sampling directions that spread them out of the viewing plane. We also demonstrated that it is important to consider the directionality of k‐space sampling as for example, streaks, ghosting, and other artifacts due to sharp discontinuities in k‐space generally manifest as artifacts perpendicular to the discontinuity. With Cartesian imaging, it is also important to consider the slowest phase encoding direction the most, as that is the one that is most at risk for severe artifacts.

In the motion magnitude experiments we showed that secondary effects (that we mostly ignored in this paper) become significant at higher magnitudes of motion, and that even small motion can produce severe artifacts. Realistic motion often involves large motion, as seen in the pediatric motion tracks used for this project, so it is essential to test novel motion correction methods under realistic conditions. While a method may reduce severe artifacts caused by small motion, this does not guarantee effectiveness for larger motion and both scenarios should be assessed when presenting new claims.

With volunteers mimicking patient motion, we further demonstrated that despite severe artifacts, blinded reviewers could still clearly distinguish specific artifact types and their appearance and orientation could be easily linked with the motion sampling plots. The appearance of the few misclassified cases (lines vs. streaks (Figure 9) and ringing vs. ghosting (Figure 10)) could be explained using the motion‐sampling plots. In further work the opposite process, predicting image artifacts from the motion sampling plots, could be explored.

### 4.1 Recommendations

Based on our findings, we propose the following best practices for evaluating motion effects and the efficacy of motion correction methods:

1. Employ motion‐sampling plots to visualize how motion states are distributed across k‐space. This aids in both interpretation and reproducibility as there is no uncertainty about which k‐space spokes were corrupted for someone replicating the results.
2. If motion experiments produce only a few discontinuities, include cases with discontinuities near the center of k‐space, which represent a worst‐case scenario.
3. Show the effect of motion corruption across different viewing planes. It is easy for directional motion artifacts to “hide” in certain planes. Ideally reviewers/readers should have access to the original data or dashboards to explore images in 3D.
4. Use realistic magnitudes of motion when testing new motion correction methods. Use motion tracks from public databases or literature.

These recommendations are intended as a starting point for a larger conversation within the MRI motion correction community in developing guidelines for motion correction focused research.

## 5 Conclusion

This study highlights the relationship between motion and sampling in neuroimaging MRI and provides methods for disseminating motion related image corruption. Using the motion‐sampling formalism we have presented a heuristic way of describing expected motion artifacts in both simple directed motion cases as well as in realistic motion.

## Supporting information


**Figure S1:** The target motion along with the volunteer performed motion for the 5 different subjects mimicking 5 different patient motions four times each for different sampling conditions. The tracks show that the volunteers managed to follow the target motion with high accuracy and similar performance for the different sampling cases.
**Figure S2:** All identified artifacts by the five reviewers displayed per subject (rows) and sampling approach (column). Cartesian sampled data are shown with yellow plots, stack‐of‐stars data in blue. The red circle shows the threshold of two reviewers identifying an artifact for it being considered in the full analysis and explained in figures S3–S7.
**Figure S3:** Subject 1 with their artifact scores and motion sampling plots.
**Figure S4:** Subject 2 with their artifact scores and motion sampling plots.
**Figure S5:** Subject 3 (same data as in Figure 9) with their artifact scores and motion sampling plots.
**Figure S6:** Subject 4 (same data as in Figure 10) with their artifact scores and motion sampling plots.
**Figure S7:** Subject 5 with their artifact scores and motion sampling plots.
**Figure S8:** Subject 1 target motion track shown in the smooth Cartesian case. The top two rows show the translation and rotation parameters. Note how closely correlated Translation X and Y is to Rotation Z and Translation Z is to Rotation X and Y as these motions inadvertently happen together when moving ones head. For the above plots, the scanner iso‐centre was used as origin, meaning that a portion of the translational components arise due to the head rotating around a point away from the iso‐centre. The bottom row shows a motion‐time plot for both translations and rotations. The bottom right plot shows total RMS displacement assuming a 64 mm radius sphere [13] as an alternative way of displaying motion on a motion‐sampling plot.

## Data Availability

Code used in this project is available for download using this link: https://gitlab.com/neuromr_karolinska/motion_sampling_plots.git (SHA: cfa3baaa). The data that support the findings of this study are openly available on Zenodo at https://zenodo.org/uploads/15697609,  https://doi.org/10.5281/zenodo.15697609.
