# Supplementary material for: An Exploration of Motion‐Sampling Interactions in 3D MRI for Neuroimaging
Source: Magn Reson Med. 2025 Nov 7;95(3):1448–61. doi: 10.1002/mrm.70132 (PMC12746353; doi:10.1002/mrm.70132)
Supplement: Supplementary file 1 — Figure S1: The target motion along with the volunteer performed motion for the 5 different subjects mimicking 5 different patient motions four times each for different sampling conditions. The tracks show that the volunteers managed to follow the target motion with high accuracy and similar performance for the different sampling cases. Figure S2: All identified artifacts by the five reviewers displayed per subject (rows) and sampling approach (column). Cartesian sampled data are shown with yellow plots, stack‐of‐stars data in blue. The red circle shows the threshold of two reviewers identifying an artifact for it being considered in the full analysis and explained in figures S3–S7. Figure S3: Subject 1 with their artifact scores and motion sampling plots. Figure S4: Subject 2 with their artifact scores and motion sampling plots. Figure S5: Subject 3 (same data as in Figure 9) with their artifact scores and motion sampling plots. Figure S6: Subject 4 (same data as in Figure 10) with their artifact scores and motion sampling plots. Figure S7: Subject 5 with their artifact scores and motion sampling plots. Figure S8: Subject 1 target motion track shown in the smooth Cartesian case. The top two rows show the translation and rotation parameters. Note how closely correlated Translation X and Y is to Rotation Z and Translation Z is to Rotation X and Y as these motions inadvertently happen together when moving ones head. For the above plots, the scanner iso‐centre was used as origin, meaning that a portion of the translational components arise due to the head rotating around a point away from the iso‐centre. The bottom row shows a motion‐time plot for both translations and rotations. The bottom right plot shows total RMS displacement assuming a 64 mm radius sphere [13] as an alternative way of displaying motion on a motion‐sampling plot. [file MRM-95-1448-s001.docx]

# Supplemental material

Motion targets (five pediatric patients) from the [brainmrimotion.org](http://brainmrimotion.org) data base The data were taken from the T1w SPGR sequence in these patients:

1. Adam vanNiekerk@CNS/KI

"AcqDate": "2022-10-28",

"StudyID": "7366",

"SubjectAge": 4,

"SubjectGender": "Male"

2. Adam vanNiekerk@CNS/KI

"AcqDate": "2022-11-25",

"StudyID": "7522",

"SubjectAge": 6,

"SubjectGender": "Female"

3. Adam vanNiekerk@CNS/KI

"AcqDate": "2022-12-02",

"StudyID": "7578",

"SubjectAge": 5,

"SubjectGender": "Male",

4. Adam vanNiekerk@CNS/KI

"AcqDate": "2023-01-20",

"StudyID": "7928",

"SubjectAge": 10,

"SubjectGender": "Female",

5. Adam vanNiekerk@CNS/KI

"AcqDate": "2023-02-03",

"StudyID": "8055",

"SubjectAge": 9,

"SubjectGender": "Male",


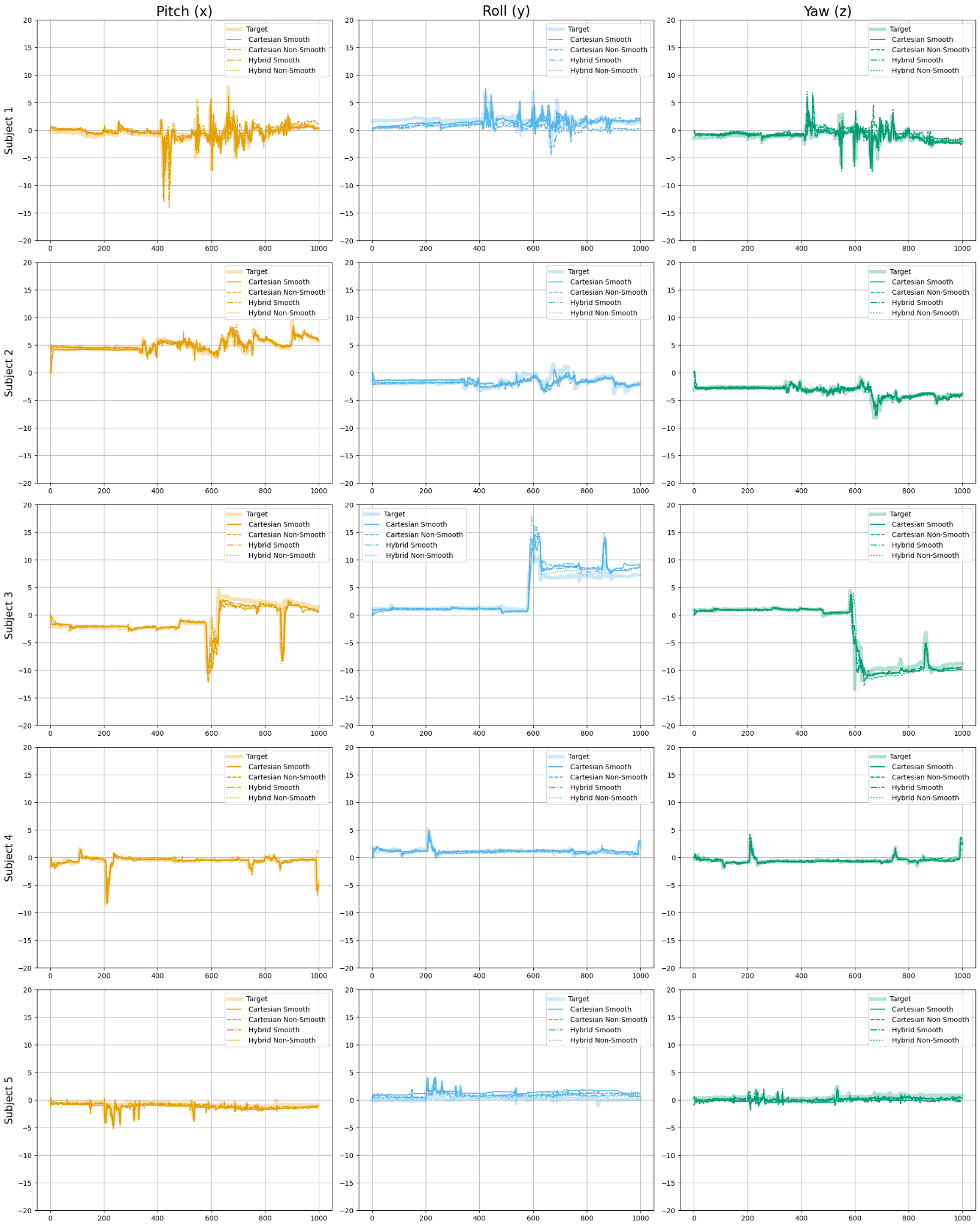


Figure S1: The target motion along with the volunteer performed motion for the 5 different subjects mimicking 5 different patient motions four times each for different sampling conditions. The tracks show that the volunteers managed to follow the target motion with high accuracy and similar performance for the different sampling cases.


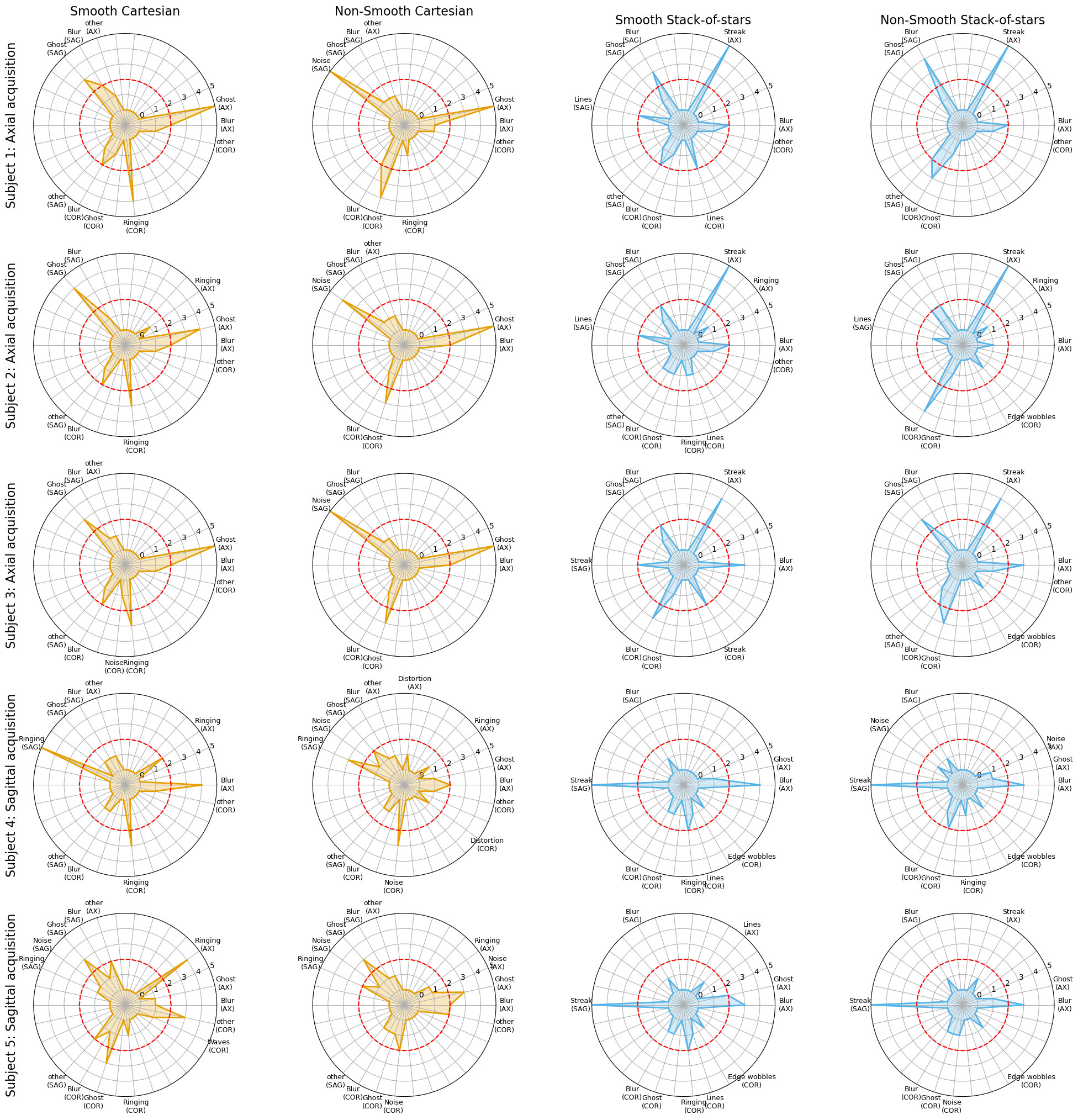


Figure S2: All identified artefacts by the five reviewers displayed per subject (rows) and sampling approach (column). Cartesian sampled data are shown with yellow plots, stack-of-stars data in blue. The red circle shows the threshold of two reviewers identifying an artifact for it being considered in the full analysis and explained in figures S3-S7.


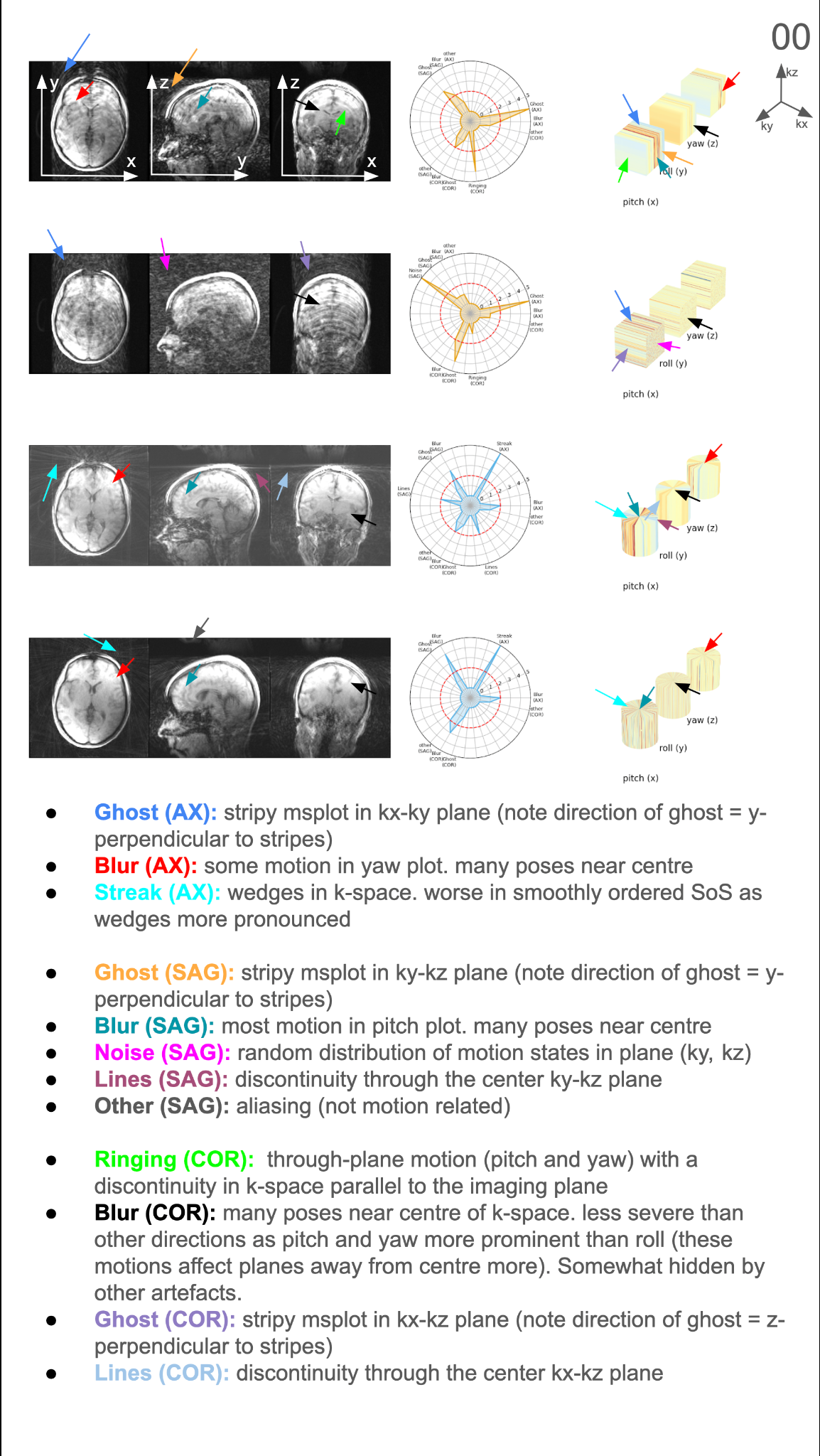


Figure S3: Subject 1 with their artefact scores and motion sampling plots.


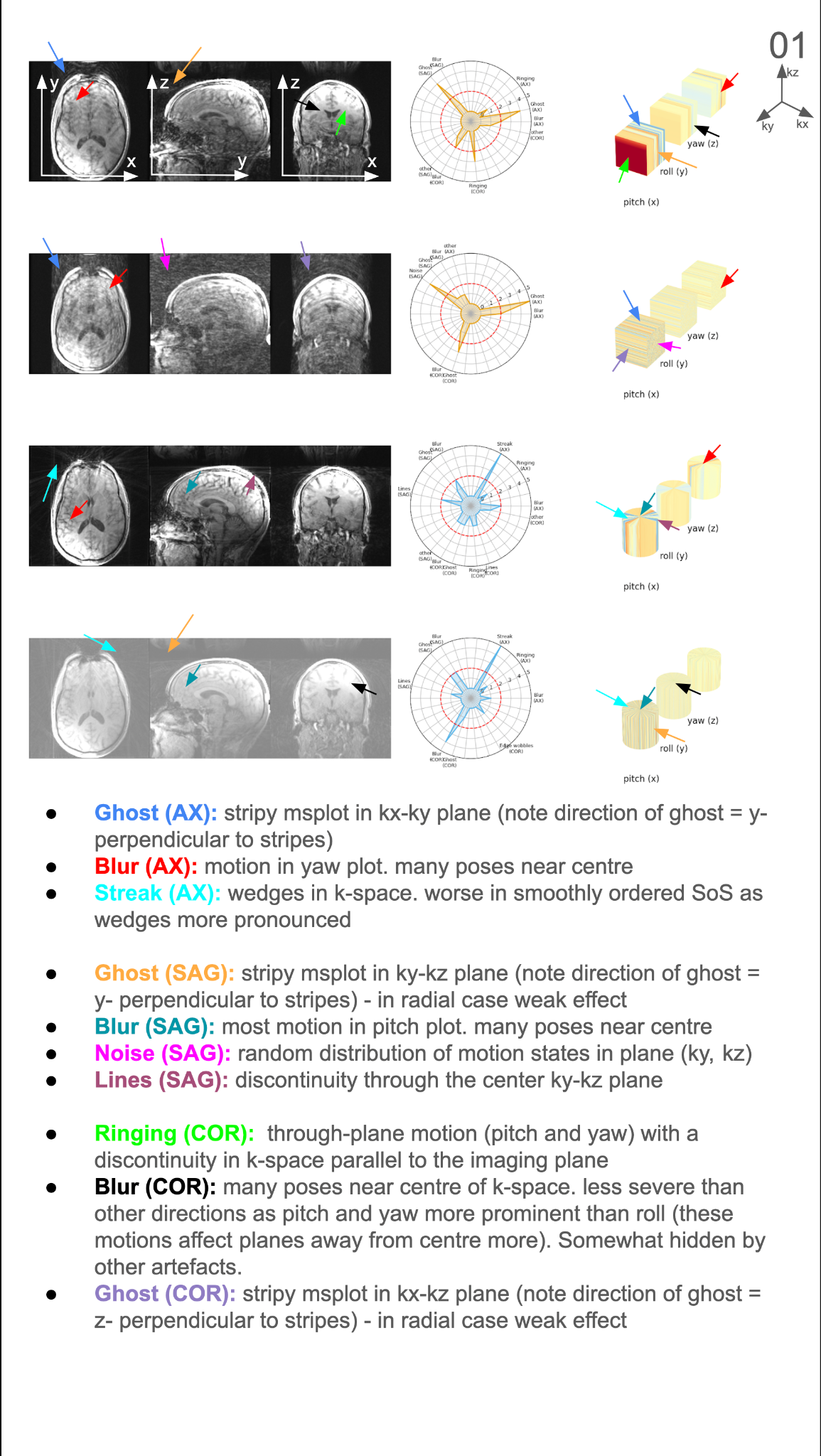


Figure S4: Subject 2 with their artefact scores and motion sampling plots.


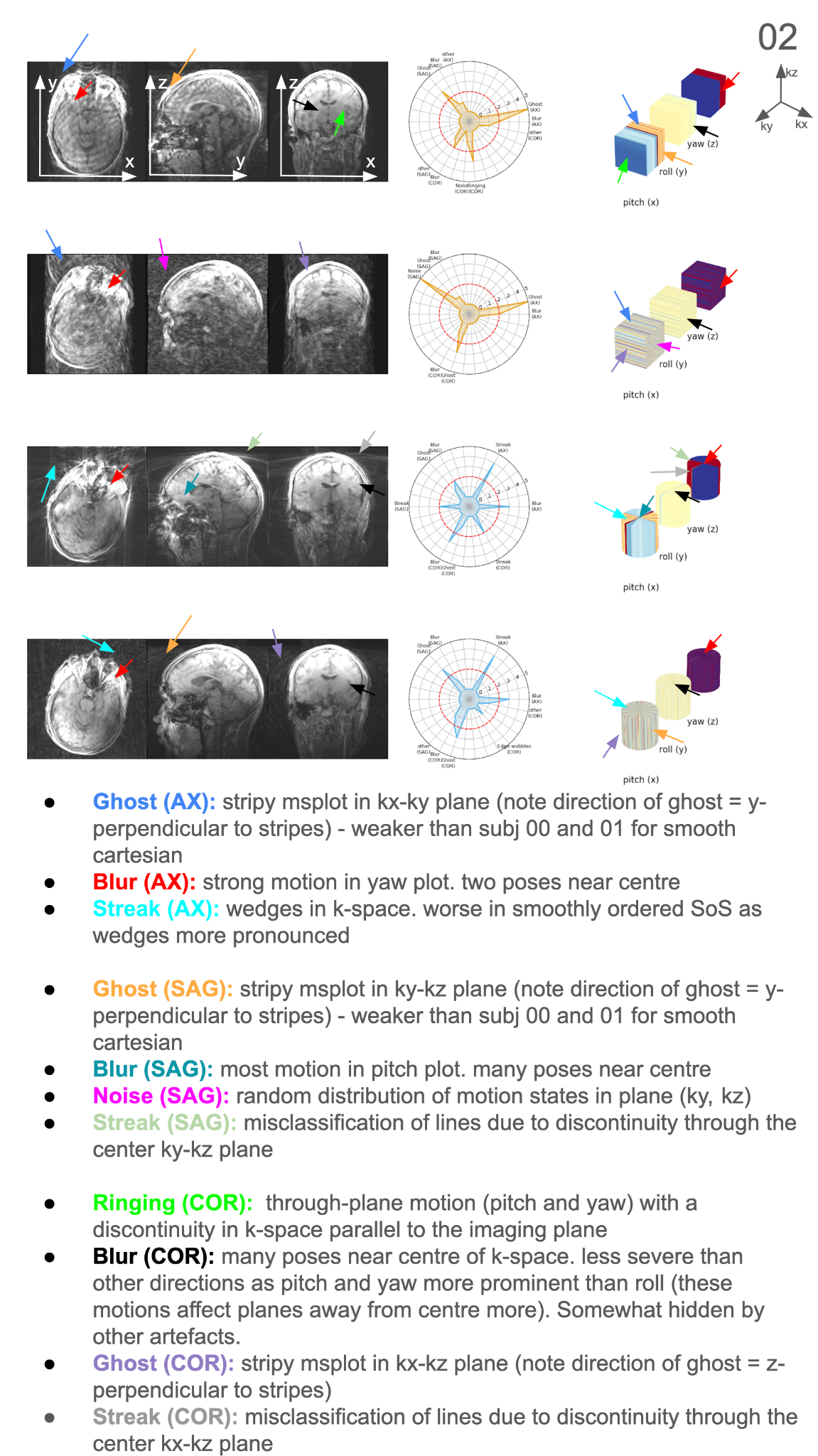


Figure S5: Subject 3 (same data as in figure 9) with their artefact scores and motion sampling plots.


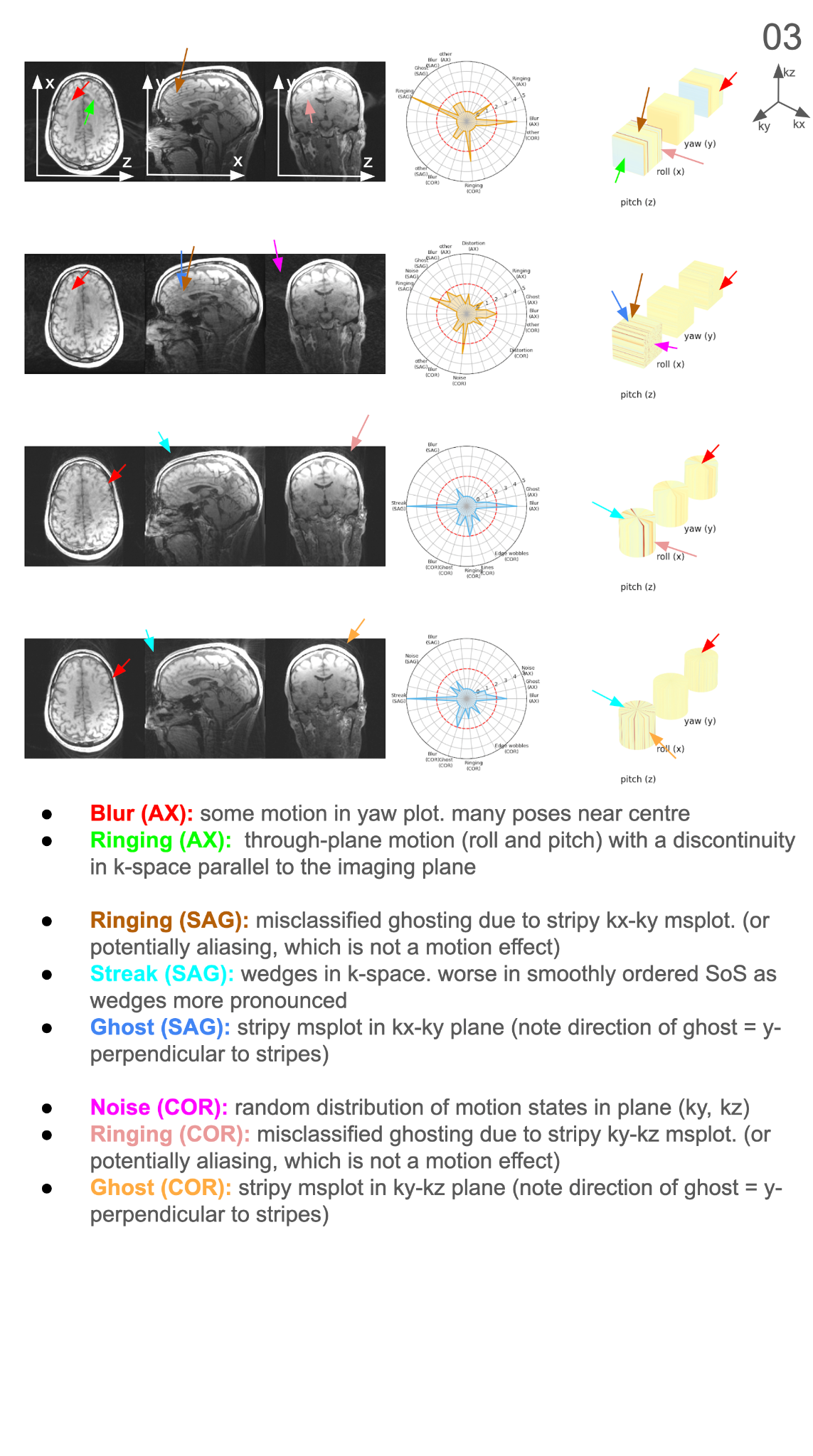


Figure S6: Subject 4 (same data as in figure 10) with their artefact scores and motion sampling plots.


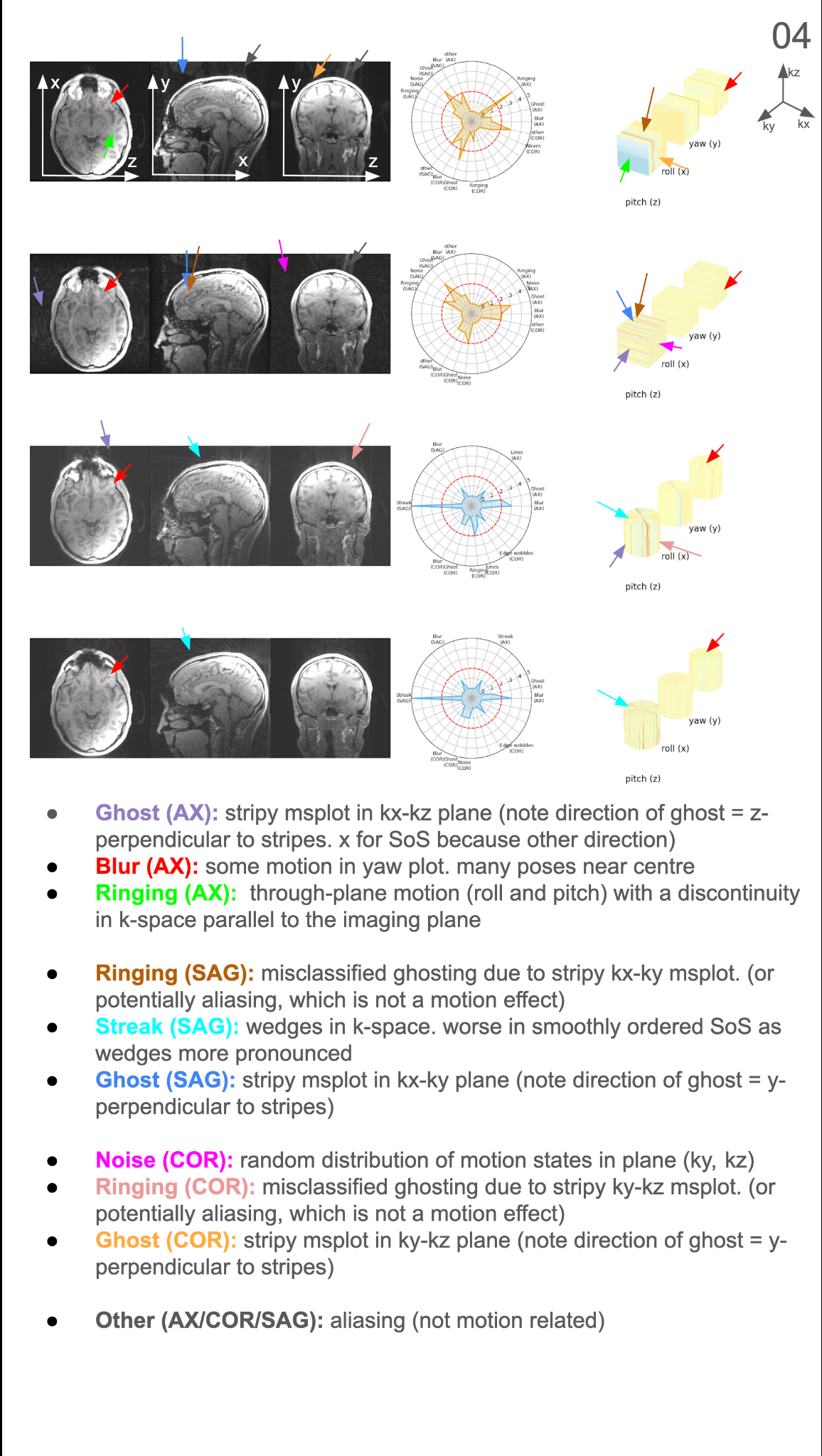


Figure S7: Subject 5 with their artefact scores and motion sampling plots.


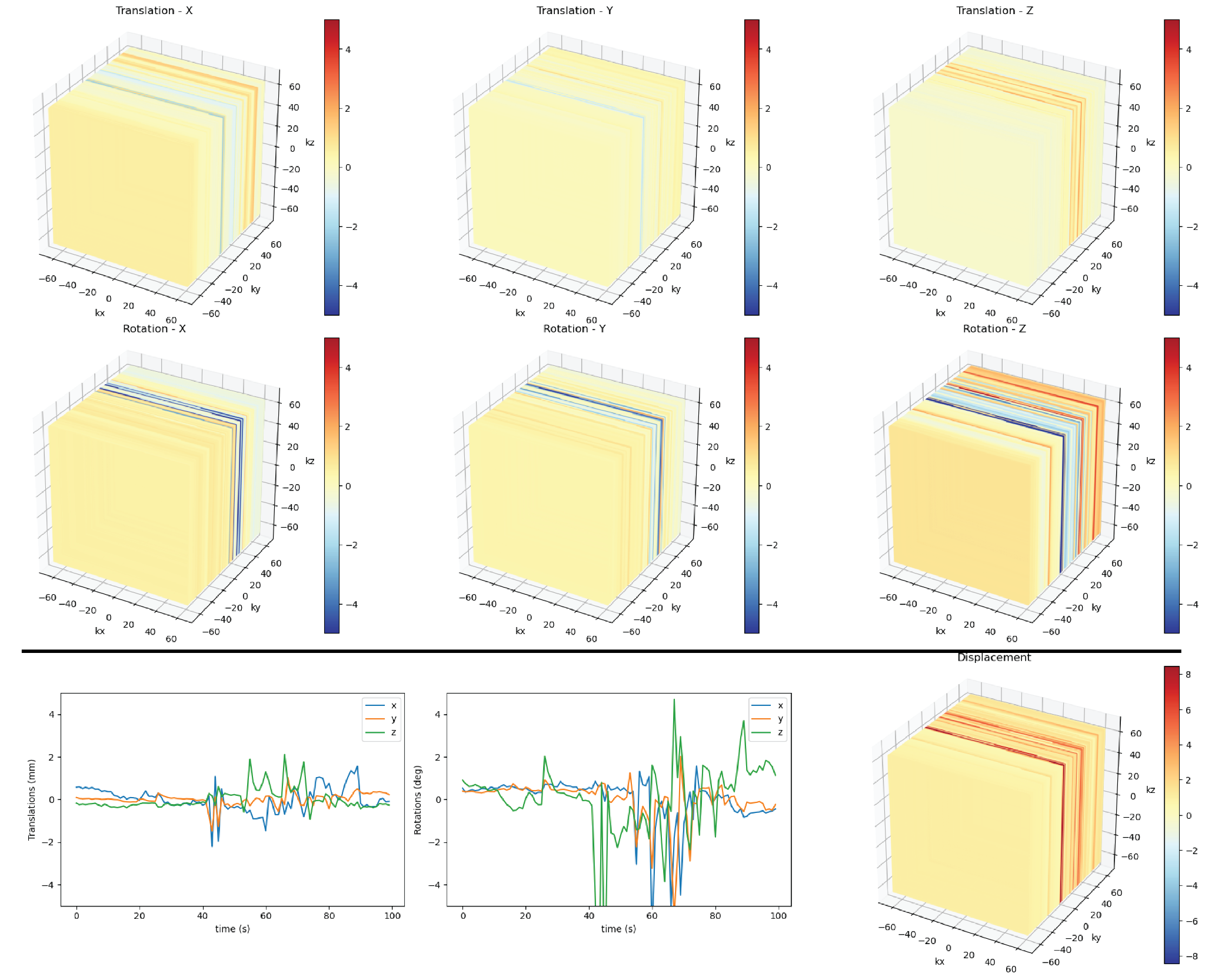


Figure S8: Subject 1 target motion track shown in the smooth Cartesian case. The top two rows show the translation and rotation parameters. Note how closely correlated Translation X and Y is to Rotation Z and Translation Z is to Rotation X and Y as these motions inadvertently happen together when moving ones head. For the above plots, the scanner isocentre was used as origin, meaning that a portion of the translational components arise due to the head rotating around a point away from the iso-centre. The bottom row shows a motion-time plot for both translations and rotations. The bottom right plot shows total RMS displacement assuming a 64 mm radius sphere[^13^](https://www.zotero.org/google-docs/?ExVkPq) as an alternative way of displaying motion on a motion-sampling plot.
